# Impact of the polymer backbone chemistry on interactions of amino-acid-derived zwitterionic polymers with cells

**DOI:** 10.1016/j.bioactmat.2023.01.005

**Published:** 2023-01-10

**Authors:** Meike N. Leiske, Bruno G. De Geest, Richard Hoogenboom

**Affiliations:** a Supramolecular Chemistry Group, Centre of Macromolecular Chemistry (CMaC), Department of Organic and Macromolecular Chemistry, Ghent University, Krijgslaan 281 S4, B-9000, Ghent, Belgium; b Department of Pharmaceutics and Cancer Research Institute Ghent (CRIG), Ghent University, Ottergemsesteenweg 460, B-9000, Ghent, Belgium

**Keywords:** Amino-acid-functionalised polymer, Zwitterionic polymer, Cell-specific polymer, Cancer-targeting, RAFT-Polymerisation

## Abstract

Zwitterionic polymers are known to interact with cells and have been shown to reveal cancer cell specificity. In this work, the importance of the chemistry of the polymer backbone for the cellular specificity of amino-acid-derived polyzwitterions is demonstrated. A series of glutamic acid (Glu)-based vinyl monomers (*i.e.*, an acrylate, a methacrylate, an acrylamide, and a methacrylamide) were prepared and used for reversible addition-fragmentation chain-transfer (RAFT) polymerisation, yielding defined polymers with narrow size distribution (Ð < 1.3). All Glu-functionalised, zwitterionic polymers revealed high cytocompatibility; however, differences in cellular association and specificity were observed. In particular, the methacrylamide-derived polymers showed high association with both, breast cancer cells and non-cancerous dendritic cells and, consequently, lack specificity. In contrast, high specificity to only breast cancer cells was observed for polyacrylates, -methacrylates, and -acrylamides. Detailed analysis of the polymers revealed differences in hydrophobicity, zeta potential, and potential side chain hydrolysis, which are impacted by the polymer backbone and might be responsible for the altered the cell association of these polymers. It is shown that a slightly negative net charge is preferred over a neutral charge to retain cell specificity. This was also confirmed by association experiments in the presence of competitive amino acid transporter substrates. The affinity of slightly negatively charged Glu-derived polymers to the xCT Glu/cystine cell membrane antiporter was found to be higher than that of neutrally charged polymers. Our results emphasise the importance of the polymer backbone for the design of cell-specific polymers. This study further highlights the potential to tailor amino-acid-derived zwitterionic materials beyond their side chain functionality.

## Highlights

• The backbone chemistry of zwitterionic polymers has a major impact on their cellular interactions.
• Amino-acid-derived zwitterionic polymers are specific to cancer cells.
• Amino-acid-derived zwitterionic polymers have low-fouling properties.
• Amino-acid-derived polymers with a slightly negative charge comprise a higher cell specificity than neutrally charged ones.

## Graphical abstract

**Figure:**
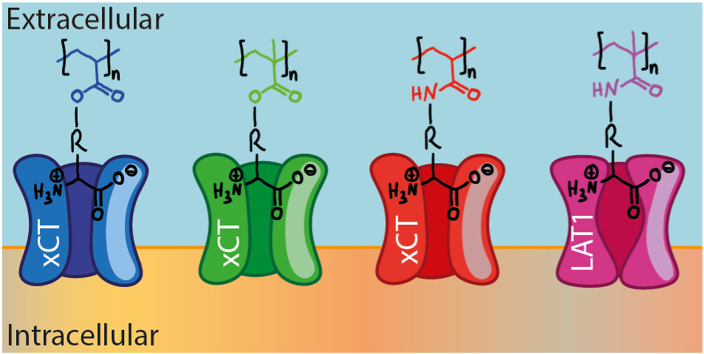

## 1 Introduction

During the last decades, polymer nanomedicine has received rising attention [1]. Synthetic polymers have been used for drug delivery platforms in different ways: (i) polymer nanoparticles, (ii) stimuli-responsive polymer drug conjugates, or (iii) therapeutic polymers [[2], [3], [4], [5], [6], [7], [8], [9]]. Independent from the aimed mechanism of delivery, a high degree of cell or tissue specificity of polymer carriers is indispensable [4]. To achieve this, hydrophilic non-ionic polymers (*e.g.*, poly(ethylene glycol)s, poly(cyclic imino ether)s, and poly(vinyl pyrrolidine)s) have gained interest due to their low protein adsorption and prolonged blood circulation time [10,11]. While low fouling properties are advantageous to decrease unwanted cellular interactions, they can also reduce the association with the target tissue and cells [12,13].

To this end, zwitterionic polymers have emerged as promising alternative as they can combine low fouling characteristics [14] with tissue specificity [15]. Previously, most studies have focussed on polybetaines (*i.e.* carboxybetaines, sulfobetaines, or phosphobetaines) [16,17]. Besides their appealing low fouling properties [16,18], many studies have observed accumulation in cancer cells and tumour tissue [[19], [20], [21], [22], [23]]. Recently, Sakurai and co-workers described the affinity of polycarboxybetaine bottle brushes to zwitterionic transporters on the cell surface [19]. While betaines are not sensitive to pH changes and, thus, comprise mostly pH-independent low fouling properties [16], in some cases these changes are assumed to be advantageous. In particular, a shift from neutral to slightly cationic charge caused by a pH drop (*e.g.* in the tumour environment or in intracellular acidic vesicles such as endosomes and lysosomes) can facilitate an increase in membrane interactions of these polymers and, consequently, enhance their cellular uptake and cytoplasmic release [21]. To this end, *α*-amino-acid-modified polymers possess interesting properties, mainly due to their dynamic pH response [24]. In addition, their chemical versatility allows an effortless manipulation of the polymer structure. Recently, several groups have independently reported cancer cell specificity of different *α*-amino-acid-functionalised polymers. The contribution of amino acid transporters (AATs) to this specificity was also verified [19,25,26]. Interestingly, different AATs have been suggested as potential targets within these studies. To this end, a direct comparison of the developed systems appears to be impossible due to significant variations within the polymer structures, *e.g.,* degree of polymerisation, side chain structure, and backbone chemistry. In addition, the hydrophobicity of polymers has been reported to impact the interactions with AATs, such as L-type amino acid transporter 1 (LAT1) [27]. It is known from other polymer characteristics, such as mechanical properties, that minor modifications in the chemistry of the polymer chain, *e.g.* by the exchange of heteroatoms, can cause significant changes to their performance [28]. However, when it comes to biomedical applications, the comparison of different polymers often appears to be difficult due to limited knowledge of material characteristics and the impact of changes to its properties on their *in vitro* (or *in vivo*) behaviour. It has recently been shown that alterations to the chemical fingerprint of non-ionic polymers alter their interaction with proteins and (immune) cells drastically due to changes in the hydrophilicity and hydration of the materials [29,30].

Within this study we demonstrate the importance of the backbone chemistry of zwitterionic amino-acid-derived polymers for their cellular specificity. To achieve this, *N*-Boc-*l*-glutamic acid *α*-*tert*-butyl ester (*N*Boc-Glu-O*t*Bu) served as the starting material to synthesise four different vinyl monomers, namely an acrylate, a methacrylate, an acrylamide and a methacrylamide, which can all be readily polymerised by reversible addition-fragmentation chain-transfer (RAFT) polymerisation to obtain narrow disperse polymers with zwitterionic glutamate (Glu)-derived side chains and systematic variations in the polymer backbone. The resulting polymers were characterised regarding their pH response, hydrolytic stability, and protein fouling. After verification of their cytocompatibility, the cellular specificity and association mechanism were investigated by means of flow cytometry. The results showed that the cellular specificity of zwitterionic polymers is highly impacted by their backbone chemistry. In addition, differences regarding the involved AATs were observed. These results underline the importance of the polymer backbone structure of zwitterionic polymers for their cellular specificity.

## 2 Experimental part

Experimental details concerning the materials and instrumentation as well as synthetic procedures for polymer synthesis can be found in the supporting information.

### 2.1 Polymer properties in aqueous environment

*Isoelectric point (IEP).* Polymers were solubilised in deionised water (diH_2_O) at a concentration of 10 mg mL^−1^. The pH was adjusted using 0.01 M NaOH and HCl solutions and measured with a generic digital pH meter. At indicated pH values, zeta potential measurements were conducted to determine the charge of the polymers.

*pD-dependent*^*1*^*H NMR measurements.* Polymers were solubilised in D_2_O at a concentration of 10 mg mL^−1^. The pD was adjusted using 0.01 M NaOD and DCl solutions and measured with a generic digital pH meter. The obtained value was corrected by 0.4 according to literature procedures [31]. Subsequently, ^1^H NMR measurements were performed at room temperature.

*Long-term stability.* Deuterated PBS was prepared by dissolving phosphate buffered saline tablets in D_2_O. Polymers were solubilised in deuterated PBS at a concentration of 10 mg mL^−1^ and stored in a heating block at 37 °C. At indicated time points, ^1^H NMR spectra (300 MHz) were recorded at room temperature.

*Interaction of polymers withbovine serum albumin (BSA).* A literature procedure was used [32]. The interaction of polymers with BSA was assessed by monitoring the *D*_h_ of its aggregates in the presence of different polymers in solution. Typically, 1 mL of 0.2 wt% of aqueous solution of BSA and 1 mL of 0.2 wt% of a respective polymer solution were prepared separately. These two solutions were then mixed in a polystyrene semimicro cuvette at 25 °C and the cuvette was placed in the DLS for monitoring the *D*_h_ of the expectedly formed polymer adsorbed BSA aggregates periodically (up to 24 h) from the time of mixing.

*Hydrophilic/hydrophobic ratio.* The procedure was modified from literature [33]. A known concentration of Glu-functionalised polymers (1 mg mL^−1^) was dissolved in phosphate-buffered saline (PBS). An equal volume of dichloromethane (DCM) was added, and the solution was vortexed for a few minutes. The solution was allowed to stand for 24 h to separate the aqueous and the organic layer. A sample was carefully taken from each layer and analysed *via* fluorescence measurements (λ_ex_ = 490 nm, λ_em_ = 520 nm). To obtain the hydrophilic/hydrophobic ratio, the raw results were normalised by the peak maximum of the emission trace of the respective polymer observed in PBS. The results are shown as normalised fluorescence intensity.

### 2.2 Biological assays

*Cell culture.* MDA-MB-231 cells were grown in DMEM/F12 supplemented with 10% (v/v) fetal bovine serum (FBS), 100 U mL^−1^ penicillin, and 100 μg mL^−1^ streptomycin. DC2.4 cells were grown in RPMI-1640 medium supplemented with 10% (v/v) FBS, 100 U mL^−1^ penicillin, 100 μg mL^−1^ streptomycin, and 1 mM sodium pyruvate. Cells were maintained at 37 °C in a fully humidified atmosphere containing 5% CO_2_.

*Cell viability.* Cells were cultured as described above. For the cell viability assay, cells (10^4^ per well) were seeded in 96-well plates and allowed to adhere overnight. No cells were seeded in the outer wells. The media was subsequently removed and replaced by fresh polymer-containing media. Then, the cells were incubated at 37 °C for an additional 24 h. After that, the media was removed, the cells were washed with 100 μL Dulbecco's PBS (DPBS), and then fresh media containing the thiazolyl blue tetrazolium bromide (MTT) (concentration: 1 mg mL^−1^) was added (100 μL per well). Note: MTT (50 mg) was dissolved in 10 mL of sterile DPBS, filtrated (membrane, 0.22 μm), and 1 to 5 diluted in culture medium prior to use in this assay. After incubation for 3 h at 37 °C, 50 μL of dimethyl sulfoxide (DMSO) were added to each well and the plates were gently shaken in the dark for 1 h to dissolve the formazan crystals. Quantification was done by measuring the absorbance at λ = 590 nm using a microplate reader. Untreated cells on the same plate served as negative control (100% viability), cells treated with 20% DMSO as positive control (0% viability), and wells without cells as background. Experiments were performed in triplicates on three different plates.

(1) $$\%\;Cell\;viability=\frac{Abs.\;sample-Abs.\;background}{Abs.\;negative\;control-Abs.\;background}∙100$$

*Cellular association.* Polymers were investigated for their association with MDA-MB-231 and DC2.4 cells *via* flow cytometry. Cells were seeded at 50,000 cells per well in 24-well microplates and cultured with 500 μL media for 24 h. The media was removed, and the cells were washed with DPBS twice. Then, 450 μL of fresh media (with or without FBS as indicated) was added and subsequently, 50 μL of polymer solution (concentration: 1 mg mL^−1^) in PBS was added individually to each well. DMEM/F12 was used as media for all cell association experiments. For assays with competitive amino acids, a 10× stock of the corresponding compound was prepared in DPBS and then mixed with cell culture media to obtain the 1× concentration. 450 μL of this mixture were used as fresh media. After incubation for predetermined times, the cells were washed with 500 μL cold DPBS and trypsinised (130 μL per well) for 5 min. Then, 500 μL of fresh media were added and the cells were centrifuged for 5 min at 350×*g*. The supernatant was discarded, and the cells were subsequently resuspended in 200 μL DPBS. Samples were analysed using a BD Accuri flow cytometer. For each sample, at least 10,000 events (P3, Fig. S18) were analysed (n = 3 in each experiment).

The mean fluorescence intensity (MFI) refers to the geometric mean value of all analysed living cells in the respective experiment. Data was processed using the FlowJo software package. For the gating strategy, please refer to Fig. S18. The relative MFI refers to a corrected value that keeps in account the fluorescent labelling efficiency of the polymer.

*Statistical analysis.* All data plotted with error bars are expressed as means with standard deviation. The P values were generated by analysing data with a one-way ANOVA and Turkey test using OriginLab.

## 3 Results and discussion

### 3.1 Synthesis and characterisation

**Scheme 1:**
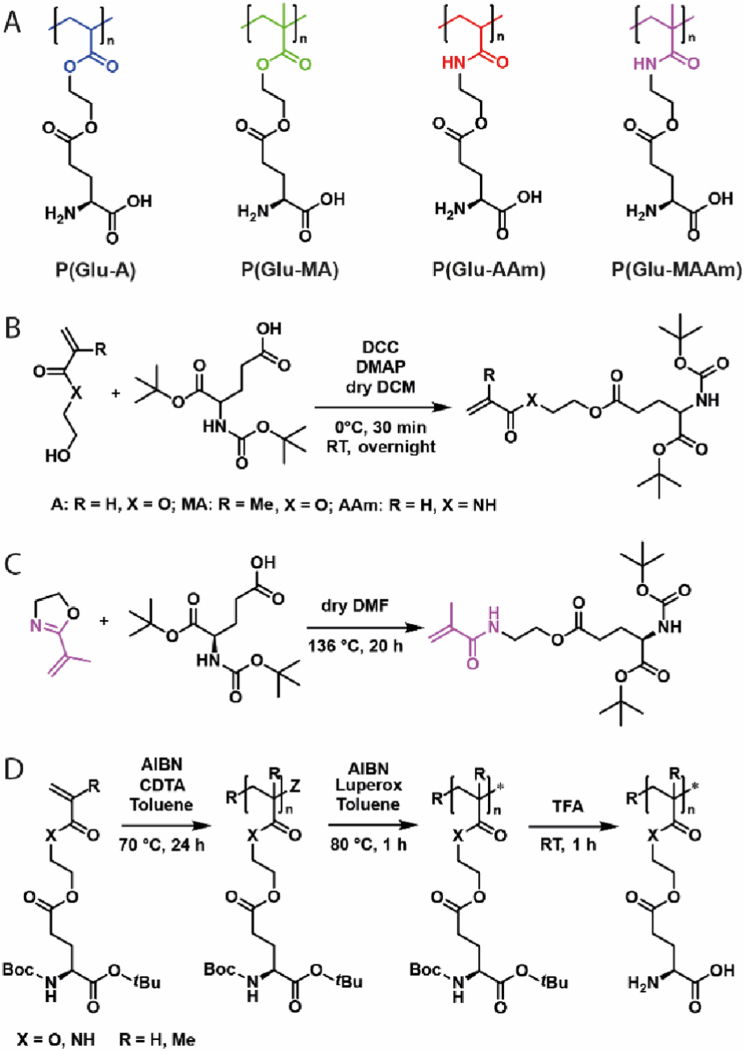
A: Structures of Glu-derived zwitterionic polymers explored within this study: P(Glu-A), P(Glu-MA), P(Glu-AAm), P(Glu-MAAm). B: Synthetic strategy for the synthesis of Glu-derived acrylates (A), methacrylates (MA), and acrylamides (AAm) *via* Steglich esterification. C: Synthesis of Glu-derived methacrylamides *via* ring-opening of 2-isopropenyl-2-oxazoline. D: Synthesis of polymers *via* RAFT-polymerisation, Z-group removal, and subsequent acidic deprotection. For simplification purposes, polymers are presented in their non-ionic form.

The aim of this project was to investigate the effect of the backbone chemistry on the cell association and specificity of zwitterionic, amino-acid-derived polymers. Hydrophilic (*e.g.*, serine), basic (*e.g.*, glutamine), or acidic amino acids (*e.g.*, glutamic acid (Glu)) may serve as starting material for the design of vinylic monomers while maintaining the *α*-amino acid functionality. Acidic amino acids can be easily conjugated to commercially available hydroxy-functionalised vinyl monomers. Inspired by its higher hydrophobicity and chain-flexibility as well as promising results during a previous study [26], Glu was chosen over aspartic acid in this study. To design various zwitterionic Glu-derived zwitterionic polymers, which solely differ in their backbone structure, the Glu-derivative 5-(*tert*-butoxy)-4-((*tert*-butoxycarbonyl)amino)-5-oxopentanoic acid (*N*Boc-Glu-O*t*Bu) was chosen as precursor for the synthesis of four different vinyl monomers: (i) *N*Boc-Glu-O*t*Bu acrylate (*N*Boc-Glu-O*t*Bu-A), (ii) *N*Boc-Glu-O*t*Bu methacrylate (*N*Boc-Glu-O*t*Bu-MA), (iii) *N*Boc-Glu-O*t*Bu acrylamide (*N*Boc-Glu-O*t*Bu-AAm), and (iv) *N*Boc-Glu-O*t*Bu methacrylamide (*N*Boc-Glu-O*t*Bu-MAAm) (Scheme 1A). To this end, *N*Boc-Glu-O*t*Bu-A, *N*Boc-Glu-O*t*Bu-MA and *N*Boc-Glu-O*t*Bu-AAm were synthesised by the straightforward one-step Steglich esterification reaction of 2-hydroxylethyl acrylate (HEA), 2-hydroxyethyl methacrylate (HEMA) or 2-hydroxyethyl acrylamide (HEAAm), respectively with the unprotected *γ*-carboxylic acid group of *N*Boc-Glu-O*t*Bu as recently reported for *N*Boc-Glu-O*t*Bu-A by our group (Scheme 1B) [26]. All three desired compounds were synthesised by this method in adequate yields of 59% (*N*Boc-Glu-O*t*Bu-A), 45% (*N*Boc-Glu-O*t*Bu-MA) and 39% (*N*Boc-Glu-O*t*Bu-AAm).

The success of the reaction and purity of the compounds was verified *via* ^1^H NMR spectroscopy of the purified monomers, showing the appearance of the typical vinyl signals at *δ* = 5.80–6.37 ppm (Figs. S1A and S2A) and the downfield shift of the methylene group adjacent to the newly formed easter bond to *δ* = 4.28 ppm. In addition, ^13^C NMR spectroscopy confirmed the success of the reaction by a downfield shift of the corresponding carbon to *δ* = 61 ppm (Figs. S1B and S2B). HR ESI-MS measurements were performed to confirm the composition of the newly synthesised monomers *N*Boc-Glu-O*t*Bu-MA and *N*Boc-Glu-O*t*Bu-AAm by displaying a peak at *m/z* = 438.2097 Da [C_20_H_33_NO_8_ + Na]^+^ and *m/z* = 423.2087 Da [C_19_H_32_N_2_O_7_ + Na]^+^, respectively.

The fourth monomer, *N*Boc-Glu-O*t*Bu-MAAm was synthesised by the ring-opening reaction of 2-isopropenyl-2-oxazoline and the unprotected γ-carboxylic acid group of *N*Boc-Glu-O*t*Bu (Scheme 1C), which yielded the desired product in adequate yield (43%). The reaction was verified by means of ^1^H NMR and ^13^C NMR spectroscopy (Fig. S3) and the purity of the product was confirmed by HR-ESI MS measurements (*m/z* = 437.2259 Da [C_20_H_34_N_2_O_7_ + Na]^+^).

**Fig. 1:**
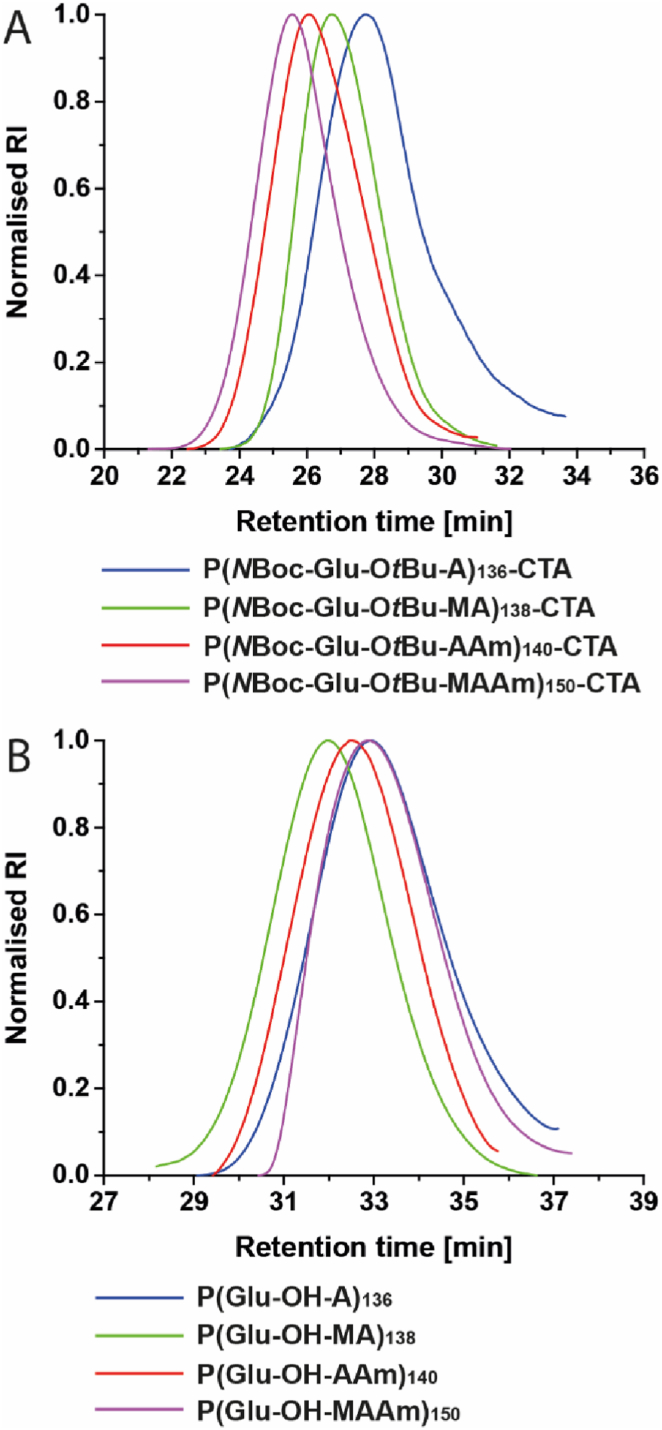
SEC traces of different zwitterionic polymers. A: SEC in DMAc. B: SEC in acetate buffer at pH 3.6 containing 30% acetonitrile and 0.1 M NaNO_3_. For molar mass distribution traces see Fig. S5 and Fig. S9. For SEC analysis after Z-group removal see Fig. S7.

After successful monomer synthesis, a small library of Glu-derived polymers with variations in the backbone chemistry was synthesised *via* RAFT polymerisation (Scheme 1D). Due to indications of the necessity of a high degree of polymerisation (DP) for cellular targeting [34], a ratio of 150:1 ([M]/[CTA]) was chosen. Despite the steric hindrance of the bulky monomers, RAFT polymerisation occurred in a controlled manner in all cases with up to 100% monomer conversion, yielding polymers of comparable DP (136–150). While larger monomers usually lead to lower conversion [35], we have previously observed similar results with amino-acid-derived monomers [26]. While the monomer concentration used for polymerisation (1.4 M) was moderately low, the high molar mass of the monomers (M > 400 g mol^−1^) may lead to an increased viscosity upon polymerisation, limiting termination events (*i.e.*, chain coupling) [36], however maintaining moderate control due to low radical concentrations in RAFT polymerisation [37]. The polymer integrity during the synthesis process was confirmed *via* SEC analysis. After synthesis and Z-group removal, SEC of all *N*Boc-Glu-O*t*Bu-derived polymers revealed monomodal distributions with narrow dispersity (Ð < 1.3) (Fig. 1A, Table S1). Differences in retention time observed for Glu-derived polymers with different backbone chemistry are related to their hydrodynamic volume, which is commonly impacted by changes to the polymer structure [38,39].

After deprotection under acidic conditions, SEC measurements on an aqueous system emphasised further the stability of all polymers (Fig. 1B). Interestingly the hydrodynamic diameter of the polymers (before and after deprotection) was altered in dependence to their polymer backbone, indicating an impact of the polymer backbone chemistry on their hydrodynamic volume in *N,N*-dimethylacetamide for the protected polymers and in aqueous environment for the zwitterionic polymers. To be able to draw conclusions about the structure property relationship of these polymers, in depth analysis of their properties in water was performed. Firstly, an acid-base titration was conducted to obtain information about the isoelectric point (IEP).

Electrophoretic light scattering (ELS) measurements (Fig. S10) provided information about the zeta potential (ζ) of the different polymers at certain pH values. It was found that P(Glu-OH-A), P(Glu-OH-AAm), and P(Glu-OH-MAAm) possess an IEP between pH 6.0 and 6.4, which is in agreement with the IEP of small molecule aliphatic amino acids, such as alanine or leucine [40]. At pH 7.4, these polymers revealed a slightly negative zeta potential (ζ ≈ −7 mV). In contrast, P(Glu-OH-MA) was found to have its IEP at pH 4.8 and, consequently, has a more distinct negative charge at pH 7.4 (ζ ≈ −20 mV). These results were also confirmed by ELS measurements in DMEM/F12 cell culture media (Table S2). Therein, only P(Glu-OH-MAAm) possessed a neutral charge, the other polymers revealed a negative charge in the following order: P(Glu-OH-MAAm) (ζ = −1.4 ± 0.3 mV) > P(Glu-OH-A) (ζ = −5.7 ± 0.3 mV) > P(Glu-OH-AAm) (ζ = −11.5 ± 0.5 mV) > P(Glu-OH-MA) (ζ = −12.6 ± 0.7 mV). The effect of the polymer backbone on polymer and gel properties has been studied before. In particular, polymers with amide-bonds possess higher rigidity than those with ester-bonds [41]. In addition, the α-methyl group in the polymer backbone of methacrylate- and methacrylamide-polymers has been found to greatly impact the ability of hydrogen bonding [42]. We assume that this combination of properties led to the distinctly different IEP and pH-responsive of P(Glu-OH-MA) when compared to the other three polymers.

In addition, dynamic light scattering (DLS) was used to gain information about a potential aggregation of the polyzwitterions at neutral charge (Figs. S11 and S12). Upon the increase of the pH value above the IEP of the polymers, DLS measurements showed aggregation of P(Glu-OH-A), P(Glu-OH-MA), and P(Glu-OH-AAm) (Figs. S11A–C), while the size of P(Glu-OH-MAAm) remained constant (Fig. S11D). The analysis of the mean count rate derived from DLS measurements further provided information about the relative particle or aggregate concentration (Fig. S12). These results showed that at the IEP of P(Glu-OH-A), P(Glu-OH-MA), and P(Glu-OH-AAm) (Figs. S12A–C) the count rate increased, while at increasing pH and, thus, negative charge of the polymers decreased, indicating a dissolution of the aggregates. In case of P(Glu-OH-MAAm), it is noteworthy to mention that solely this polymer was turbid in solution (Fig. S13) at temperatures ranging from 4 °C to 90 °C (data not shown) with a dependence on the pH value and, thus, the IEP of the polymer. An increased hydrophobicity of this polymer compared to the acrylate-, methacrylate-, and acrylamide-derived polyzwitterions was confirmed by the determination of the hydrophilic/hydrophobic ratio of fluorescently labelled polymers (Fig. S14). No fluorescence was detected from the organic (hydrophobic) phase of F-P(Glu-OH-A), F-P(Glu-OH-MA), and F-P(Glu-OH-AAm); however, a weak signal of F-P(Glu-OH-MAAm) was observed, confirming the increased hydrophobicity of this polymer. These results are in agreement with literature, reporting the isoelectric point of (poly)zwitterions as their most hydrophobic state [43,44]. Unfortunately, due to poor solubility of all polymers in organic, hydrophobic media, no quantification of the fluorescence in hydrophobic solvent was possible.

**Fig. 2:**
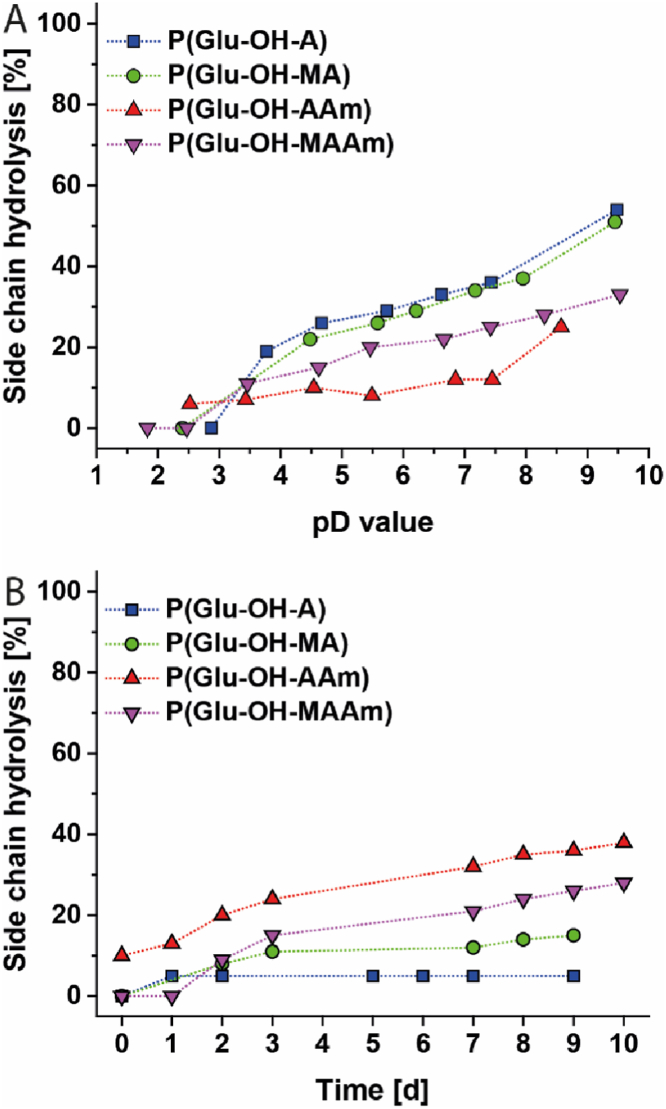
Side chain hydrolysis of zwitterionic polymers with different backbone structure as determined by ^1^H NMR measurements. A: pD-dependent hydrolysis in D_2_O. pD-adjustments were achieved by addition of 0.01 M NaOD solution. Corresponding spectra can be found in Fig. S15. B: Time-dependent hydrolysis in deuterated PBS. Samples were stored at 37 °C in-between measurements. Corresponding spectra can be found in Fig. S16.

To further investigate the differences observed regarding size and charge of polymers at different pH conditions, the stability of polymers in aqueous media was studied. Due to the potentially labile ester bond in the side chain, which connects the zwitterionic Glu units to the polymer backbone, the potential hydrolysis of these polymers was analysed by means of ^1^H NMR spectroscopy using deuterated reagents. Firstly, the pD-dependent hydrolysis was evaluated (Fig. 2A, Fig. S15). An increase of the side chain hydrolysis with increasing pH value was observed for all polymers. However, differences were observed for the polymers with different backbone chemistry. As expected, (meth)acrylate-derived polymers were found to be more prone to hydrolysis (∼30% at pH 7.4) than polymethacrylamides (∼20% at pH 7.4) or polyacrylamides (∼10% at pH 7.4).

However, a correlation between the degree of hydrolysis and the observed zeta potential was not found. It was consequently assumed that the hydrolysis of the zwitterionic, Glu-derived polymers studied predominantly yielded polymers with hydroxyethyl side chains rather than carboxylic acids. While these results already indicated important differences, which are derived from the polymeric backbone, the effect of a buffered system on the polymer stability was expected to be more relevant to cell culture conditions. For this reason, time-resolved measurements in a buffered system (deuterated PBS, Fig. 2B, Fig. S16) were performed. Interestingly, the trend observed under non-buffered conditions was not confirmed. To this end, it is assumed that the stability of all polymers in buffered systems is enhanced compared to non-buffered systems. In addition, the degree of hydrolysis was found to be dependent on the backbone chemistry of the investigated polymers: P(Glu-OH-AAm) > P(Glu-OH-MAAm) > P(Glu-OH-MA) > P(Glu-OH-A). It is assumed that this unexpected trend is correlated to the amide-group close to the polymer backbone, which facilitates hydrogen bonding with the zwitterionic group in the side chain and, thus, can enhance side chain hydrolysis.

The lag-time in hydrolysis of P(Glu-OH-MAAm) is potentially attributed to its hydrophobic character and, consequently, slower deprotonation of the α-amino acid moiety, which can also be confirmed by the delayed upfield shift of H11 (*δ* ≈ 3.7–3.9 ppm). Noteworthy, the degree of hydrolysis of all polymers remained below 15% within one day of incubation at 37 °C, rendering them suitable for *in vitro* testing.

#### 3.1.1 Cellular interactions of different Glu-derived polymers

Before studying the cell association of Glu-derived polymers, their cytocompatibility was confirmed by an MTT assay (Fig. S17), showing high tolerance to all polymers after 24 h incubation time (CC_50_ = 5.4–6.0 mg mL^−1^, Table S2). The cytotoxic effect at higher polymer concentration might derive from the charges of the polymer, however, a tolerance of up to 5 mg mL^−1^ is far above commonly tested concentrations [45,46], thus, proving high cytocompatibility. For subsequent cell association experiments, a polymer concentration of 0.1 mg mL^−1^ was chosen and consequently no harm from the polymers to the cells was expected.

**Fig. 3:**
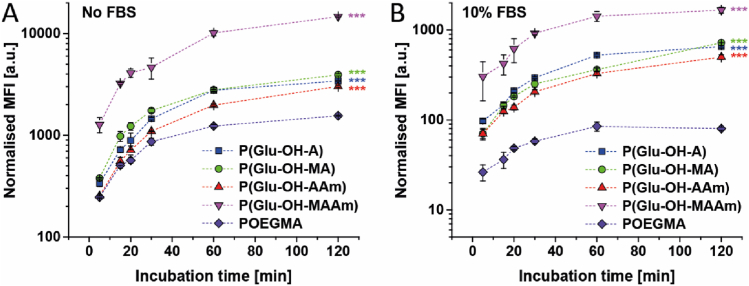
Cellular association of different fluoresceine-labelled zwitterionic polymers and POEGMA as non-ionic control determined *via* flow cytometry measurements. Polymer concentration: 0.1 mg mL^−1^. Incubation for indicated time points at 37 °C. 50,000 cells per well in 500 μL of media (24-well plate). A: Association of polymers with MDA-MB-231 breast cancer cells in DMEM/F12 starvation media. B: Association of polymers with MDA-MB-231 breast cancer cells in DMEM/F12 supplemented with 10% FBS. Statistical significance was analysed by one-way ANOVA with Tukey's test and represents significances in comparison to the non-ionic control POEGMA. ***p < 0.0005.

In an initial experiment, the cellular association of fluorescein labelled Glu-derived polymers to MDA-MB-231 breast cancer cells was studied in comparison to each other and to poly(oligoethylene glycol methacrylate) (POEGMA), a non-ionic stealth polymer (Fig. 3, Figs. S19–S23). Fluorescently labelled polymers were prepared using a fluorescein-modified chain-transfer agent (Fig. S24) to minimise differences in labelling efficiency. Time-resolved cell association kinetics were performed in starvation media (Fig. 3A) and full media containing 10% FBS (Fig. 3B). In all cases, the mean fluorescence intensity (MFI) of the cells increased over time, as expected. Noteworthy, the association of POEGMA was significantly lower (p < 0.0005) than P(Glu-OH-A), P(Glu-OH-MA), and P(Glu-OH-AAm), featuring a more distinct difference with increasing incubation time. P(Glu-OH-MAAm) revealed the highest cell association to MDA-MB-231, which was twenty times higher than POEGMA (p < 0.0005) and 2.5 times increased compared to the other studied Glu-derived polymers.

Noteworthy, the presence of FBS in the cell culture media did not reduce the cell association of zwitterionic polymers as drastically as POEGMA. For this reason, it was assumed that these polymers possess low fouling characteristics as recently reported by Mandal et al. for a similar system [32]. Low unspecific protein fouling was confirmed by DLS measurements (Fig. S25). While high fouling polymers, generally cause an aggregation of polymers and proteins in solution over time [32], no unspecific protein aggregation was observed for the polymers presented in the current study.

**Fig. 4:**
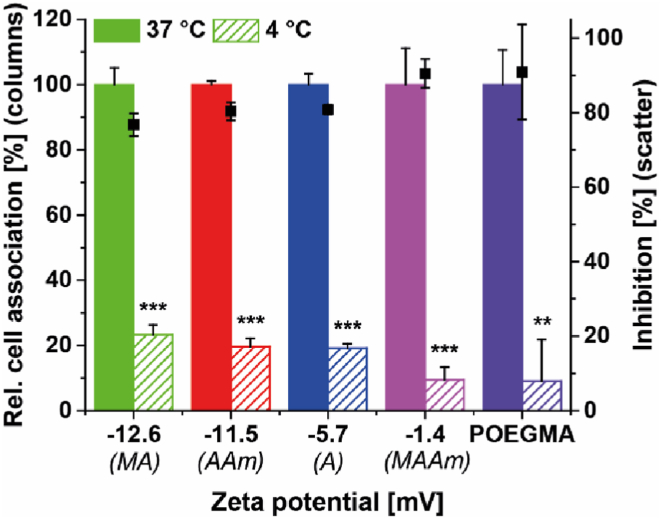
Temperature-dependent cell association of different Glu-derived polymers relative to POEGMA as non-ionic control. MDA-MB-231 breast cancer cells were incubated with indicated polymers (concentration 0.1 mg mL^−1^) at 37 °C or 4 °C in DMEM/F12 supplemented with 10% FBS for 2 h. Relative cell association was determined by normalisation of MFI of cells incubated at 37 °C. Statistical significance was analysed by one-way ANOVA with Tukey's test. ***p < 0.0005; **p < 0.005.

In addition to protein fouling, also the specificity of the observed cellular interaction was studied to obtain information about the nature of cell interactions of different zwitterionic polymers. Firstly, temperature-dependent cell association experiments were conducted to verify active polymer uptake (Fig. 4, Fig. S26). While the cellular metabolism at 37 °C is assumed to be normal, at 4 °C active, adenosine triphosphate (ATP)-dependent uptake pathways are inhibited [47]. For this reason, a comparison of the cell association at these two temperatures can provide important information about the uptake pathway (active or passive) of polymers. The cellular association of all Glu-derived polymers was inhibited significantly at 4 °C (p < 0.0005), suggesting mainly active uptake pathways, which are often upregulated in cancer cell lines and facilitate enhanced cell growth [48,49]. In addition, it was found that with lower zeta potential, the temperature-mediated decrease of cell association is less pronounced. Different groups have previously reported the passive diffusion of carboxylated (anionic) polymers through the cell membrane [50,51]. In these studies, it was claimed that a delocalised lipophilic charge (attributed to an incorporated cyanine dye) contributes to this phenomenon. It was also shown that these polymers reveal a higher cell association than zwitterionic polyphosphobetaines [51]. We assume that the slight differences in temperature-mediated suppression of cell association in this study could be triggered by similar phenomena, which are caused by the balance of anionic and cationic charges in the Glu-derived polymers. However, differences of the studied systems were less pronounced as for the reported systems and further detailed investigations on these results were outside the scope of the current study.

**Fig. 5:**
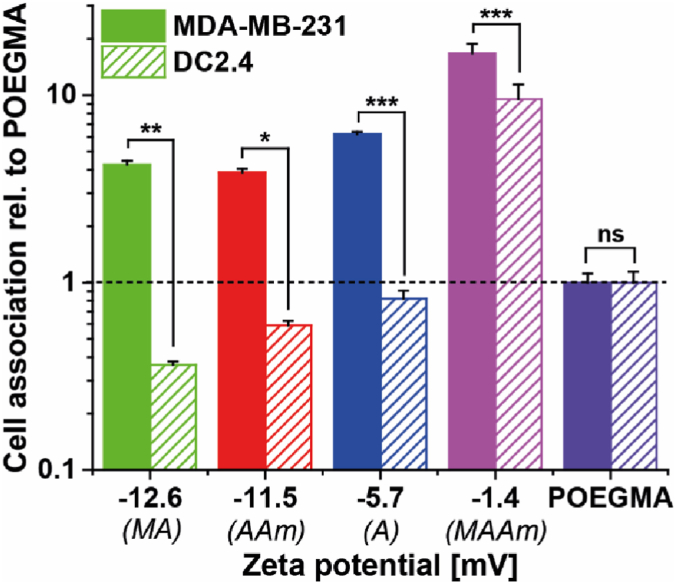
Cell association of different Glu-derived polymers relative to POEGMA as non-ionic control. MDA-MB-231 breast cancer cells and DC2.4 fibroblasts were incubated with indicated polymers (concentration 0.1 mg mL^−1^) at 37 °C in DMEM/F12 supplemented with 10% FBS for 1 h. Relative cell association was determined by normalisation of MFI of cells incubated with various polymers relative to MFI of cells incubated with POEGMA. Statistical significance was analysed by one-way ANOVA with Tukey's test. ***p < 0.0005; **p < 0.005; *p < 0.05; ns not significant at p < 0.05. For MFI histograms of DC2.4 fibroblasts, refer to Fig. S27.

To further investigate whether the increased cellular association of Glu-derived polymers to MDA-MB-231 breast cancer cells was facilitated by unspecific cell interactions or targeted cell specificity, non-cancerous DC2.4 dendritic cells were chosen as a control cell line. To exclude potential effects of the cell culture media on the polymer properties and cellular interactions, all cell association experiments were conducted using DMEM/F12. Fig. 5 shows the relative cell association of Glu-derived polymers with MDA-MB-231 breast cancer cells and DC2.4 dendritic cells in comparison to the non-ionic control polymer POEGMA. Here, a significantly decreased association with DC2.4 compared to MDA-MB-231 was observed for all Glu-derived polymers, except for P(Glu-OH-MAAm) that showed a very high association with both cell lines, suggesting hydrophobic interactions with the cell membrane, which reduce the specificity of the polymer to cancer cells. In contrast, the cell association of P(Glu-OH-A), P(Glu-OH-MA), and P(Glu-OH-AAm) to DC2.4 was lower than POEGMA. The increased hydrophobicity of P(Glu-OH-MAAm) compared to the acrylate, methacrylate and acrylamide polymer (Fig. S14), was assumed to be the driving force for this lack in cellular specificity.

In previous research, highly hydrophilic polymers have been established as anti-fouling materials with high hydration and negligible association to biological matter like proteins or cells [10,29,30,52]. On the contrary, less hydrophilic polymers showed higher association to cells, caused by hydrophobic interactions with the lipidic double layer of the cell membrane [[53], [54], [55]]. Around their IEP, zwitterionic polymers are at their most hydrophobic state as attractive forces between the polyions dominate [16,56]. However, a correlation of the zeta potential of these polymers at physiological conditions further revealed that a negative net charge of the *α*-amino acid functionalised polymers reduced the association to DC2.4 dendritic cells, potentially due to a reduction of the interaction with the negatively charged cell membrane [57]. Interestingly, compared to the non-ionic, low-fouling polymer POEGMA, the association to MDA-MB-231 breast cancer cells was enhanced despite the low net charge of P(Glu-OH-A), P(Glu-OH-MA) and P(Glu-OH-AAm). Previous studies have also reported the effect of charges on nanoparticle surfaces on cell interactions and specificity [58,59]. These results suggest that the cell association of those three polymers is mainly driven by targeted interactions with components on the cell surface of cancer cells (*i.e.* receptors or transporters), which has already been proposed for similar polymers by previous research of different groups [20,25,26,34]. To further evaluate this assumption, the cell association of polymers in the presence of competitive substrates for amino acid transporters was studied.

#### 3.1.2 Transporter specificity

**Fig. 6:**
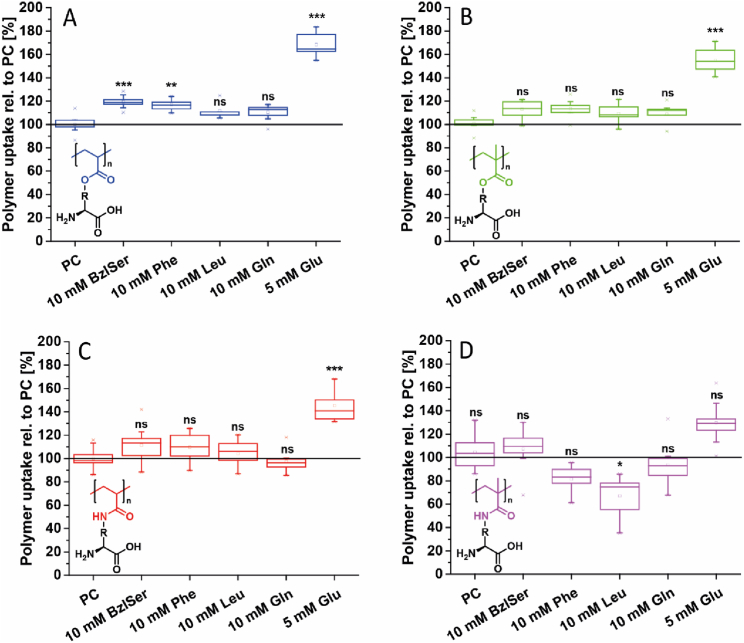
Relative cellular association of different fluoresceine-labelled zwitterionic polymers with MDA-MB-231 breast cancer cells in the absence and presence of competitive amino acids determined *via* flow cytometry measurements. PC: No competitive amino acid was used. Polymer concentration: 0.1 mg mL^−1^. Incubation for 1 h at 37 °C. 50,000 cells per well in 500 μL of DMEM/F12 + 10% FBS (24-well plate). Statistical significance was analysed by one-way ANOVA with Tukey's test. ***p < 0.0005; **p < 0.005; *p < 0.05; ns not significant at p < 0.05. A: P(Glu-OH-A). B: P(Glu-OH-MA). C: P(Glu-OH-AAm). D: P(Glu-OH-MAAm).

In our previous study, inhibitor experiments with *O*-benzyl-*L*-serine (BzlSer), a competitive inhibitor for different AATs (*e.g.*, ASCT2, LAT2, SNA2, and EEATs) [[60], [61], [62]], already suggested an involvement of AATs in the cellular association of P(Glu-OH-A) to HCC1806 breast cancer cells [26]. However, the use of more specific inhibitors to obtain further information remained unsuccessful. For this reason, the cell association of the newly synthesised polymers in the presence of different competitive substrates was investigated with MDA-MB-231 in this study (Fig. 6, Figs. S28–S34).

While we previously found that BzlSer reduced the association of P(Glu-OH-A) with HCC1806 significantly [26], these results were not confirmed with MDA-MB-231. In fact, the addition of BzlSer to the cell association experiment led to a significant increase of the association of P(Glu-OH-A) with MDA-MB-231 to up to 119 ± 5% (p < 0.0005) compared to the positive control (PC, no competitive inhibitor). For P(Glu-OH-MA), P(Glu-OH-AAm) and P(Glu-OH-MAAm) the presence of BzlSer only led to a minor increase of cell association (MA: 113 ± 8%; AAm: 111 ± 16%; MAAm: 107 ± 18%), which was not significant at p 0.05. In contrast, the presence of BzlSer did not impact the cell association of the non-ionic control polymer POEGMA (Fig. S28) and, thus, effects of the competitive inhibitor on the cell association are assumed to be attributed to the chemistry of the herein studied zwitterionic polymers. It has been previously shown that BzlSer as a therapeutic agent has a more distinct effect on the growth of HCC1806 than on that of MDA-MB-231 [60].

**Scheme 2:**
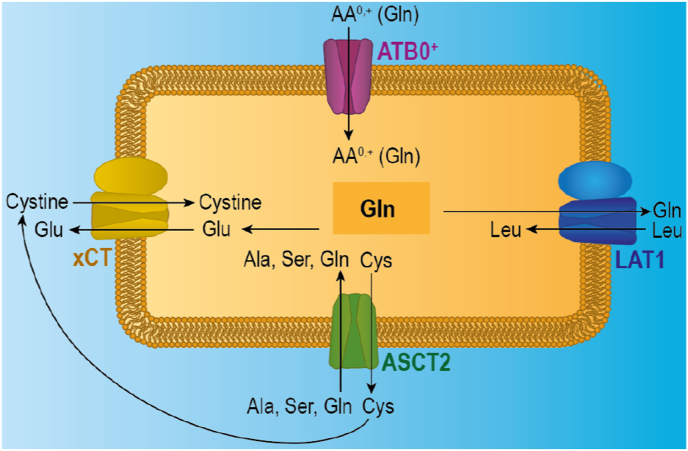
Simplified overview of the synergies of chosen amino acid transporters. Ions and further metabolic pathways have been omitted due to simplification purposes.

It has also been demonstrated previously that BzlSer can inhibit a range of amino acid transporters, including EEAT and GLYT transporters, SNAT1, SNAT2, LAT1, LAT2, as well as ASCT1 and ASCT2 [[60], [61], [62]]. Furthermore, the inhibition of certain transporters leads to starvation of the cell and, thus, a potential overcompensation through other transporters, which complicates the evaluation process [60,63]. For this reason, an excessive amino acid supply of selected amino acids (*i.e.* *L*-Phe, *L*-Leu, *L*-Gln, *L*-Glu) was chosen as strategy to further investigate potential AAT interactions of the polymers explored within this study [25]. It is noteworthy to mention that cellular nutrients such as amino acids and, consequently, their uptake transporters act in a synergistic manner as visualised in the simplified Scheme 2.

Consequently, an oversupply of *L*-Gln may lead to an increased activity of xCT to excrete the newly intracellularly synthesised *L*-Glu catalysed by the enzyme glutaminase [64]. These processes were considered for the further evaluation of the effect of competitive amino acids on the cell association of Glu-derived, zwitterionic polymers. Interestingly, the addition of *L*-Glu to the incubation media, led to a significant increase of cell association of all Glu-derived polymers (Fig. 6, Figs. S29–S32). To this end, P(Glu-OH-A) featured a cell 1.7 ± 0.1-fold cell association compared to the positive control (p < 0.0005), while P(Glu-OH-MA) showed a 1.6 ± 0.1-fold (p < 0.0005) and P(Glu-OH-AAm) a 1.4 ± 0.1-fold (p < 0.005) increase of MFI. For P(Glu-OH-MAAm) the increase in MFI was not significant, with a slight increase to 130 ± 18%. It is assumed that the increased cell association of Glu-derived polymers is facilitated by an enhanced interaction with the xCT amino acid transporters, which generally contrives the antiport of Glu and cystine (Cys-Cys) [65], the rate-limiting compound for the synthesis of glutathione (GSH). Glu is usually transported out of the cell, while Cys-Cys is transported inside [66].

Importantly, Cys-Cys comprises its IEP at pH ∼4.8 [67], leading to a negative net charge of the dimer at physiological conditions. For this reason, it is assumed that P(Glu-OH-A), P(Glu-OH-MA), and P(Glu-OH-AAm) can bind to xCT to a certain extent, which can enhance their association and uptake efficiency. In addition, the effect of sulfasalazine, an inhibitor for xCT [68], on the association of polymers was studied (Fig. S34). Interestingly, the association of polymers to MDA-MB-231 in the presence of sulfasalazine was not reduced. These results suggest that multiple AATs might be involved in the association process, potentially due to the lack of substrate-specificity of most AATs [[69], [70], [71]]. These results further point out the complexity of the studied systems as well as carefulness to be applied for the interpretation of the results. The addition of Phe and Leu as competitive substrates only led to a reduction in the MFI of P(Glu-OH-MAAm) (p_Leu_ < 0.05), suggesting an involvement of LAT1 in the association mechanism, while the other three polymers did not reveal a reduction in MFI in the presence of Leu and Phe. This difference might be associated to the hydrophobicity of P(Glu-OH-MAAm) and its neutral zeta potential in DMEM/F12 (Table S2). Similar findings have been observed for other hydrophobic polymers [27].

## 4 Conclusions

Within this study, the often-neglected impact of the polymer backbone on cellular specificity of amino-acid-derived zwitterionic polymers was studied. A small library of Glu-derived monomers, namely an acrylate (A), a methacrylate (MA), an acrylamide (AAm), and a methacrylamide (MAAm), was synthesised and polymerised *via* RAFT polymerisation. After acidic deprotection, zwitterionic, Glu-derived polymers with different backbone chemistry were obtained: P(Glu-OH-A), P(Glu-OH-MA), P(Glu-OH-AAm), and P(Glu-OH-MAAm). These polymers were studied regarding their physicochemical properties and interaction with cells. It was found that the nature of the polymer backbone impacts the pH-responsiveness (*i.e.*, pKa and IEP) of the polymers as well as their hydrophilicity and stability against hydrolysis in aqueous media. *In vitro*, it was found that all four polymers comprise a higher association with MDA-MB-231 cancer cells than a non-ionic poly(oligoethylene glycol) control polymer (POEGMA). In addition, the association of zwitterionic polymers with an acrylate, methacrylate, or acrylamide backbone to non-cancerous cells (DC2.4) was lower than that of POEGMA; however, this was not the case for the zwitterionic methacrylamide-derived polymer. Upon the addition of competitive substrates for various amino acid transporters, it was found that the polymer backbone, and consequently altered net charge of the polymers, impacts the cell and transporter affinity of these zwitterionic polymers. This comparative study demonstrates the necessity to consider characteristics of polymeric materials beyond their side chain functionalities when it comes to applications in biological environments. While the presented results are an important first step for the evaluation and application of amino-acid-derived zwitterionic polymers, certain problems need to be addressed in the future. For a better understanding of these systems, an expansion to cell lines of different origin will be indispensable. This might also help to improve the understanding of the cellular interactions. In addition, more relevant culture conditions need to be carefully examined (*e.g.*, co-culture or dynamic cell culture). Future studies will also focus on the effects of further changes to the polymer system (*e.g.*, polymer architecture) to tailor the effectiveness of cellular targeting. The detailed understanding of the structure-property-relationships and their performance in biologically relevant environments will help to optimise the design of polymer-based nanomedicines.

## CRediT authorship contribution statement

**Meike N. Leiske:** Conceptualization, Methodology, Investigation, Writing – original draft, Visualization. **Bruno G. De Geest:** Methodology, Writing – review & editing. **Richard Hoogenboom:** Conceptualization, Methodology, Writing – review & editing, Funding acquisition.

## Declaration of competing interest

There are no conflicts to declare.

## References

[1] Englert C., Brendel J.C., Majdanski T.C., Yildirim T., Schubert S., Gottschaldt M., Windhab N., Schubert U.S. (2018). Pharmapolymers in the 21st century: synthetic polymers in drug delivery applications. Prog. Polym. Sci..

[2] Duncan R., Vicent M., Greco F., Nicholson R. (2005). Polymer–drug conjugates: towards a novel approach for the treatment of endrocine-related cancer. Endocr. Relat. Cancer.

[3] Duncan R., Gaspar R. (2011). Nanomedicine(s) under the microscope. Mol. Pharm..

[4] Duncan R., Vicent M.J. (2013). Polymer therapeutics-prospects for 21st century: the end of the beginning. Adv. Drug Deliv. Rev..

[5] Greco F., Vicent M.J. (2009). Combination therapy: opportunities and challenges for polymer–drug conjugates as anticancer nanomedicines. Adv. Drug Deliv. Rev..

[6] Greish K., Fang J., Inutsuka T., Nagamitsu A., Maeda H. (2003). Macromolecular therapeutics. Clin. Pharmacokinet..

[7] Maeda H., Bharate G.Y., Daruwalla J. (2009). Polymeric drugs for efficient tumor-targeted drug delivery based on EPR-effect. Eur. J. Pharm. Biopharm..

[8] Maeda H., Seymour L.W., Miyamoto Y. (1992). Conjugates of anticancer agents and polymers: advantages of macromolecular therapeutics in vivo. Bioconjugate Chem..

[9] Maeda H. (2017). Polymer therapeutics and the EPR effect. J. Drug Target..

[10] Knop K., Hoogenboom R., Fischer D., Schubert U.S. (2010). Poly(ethylene glycol) in drug delivery: pros and cons as well as potential alternatives. Angew. Chem., Int. Ed..

[11] Barz M., Luxenhofer R., Zentel R., Vicent M.J. (2011). Overcoming the PEG-addiction: well-defined alternatives to PEG, from structure–property relationships to better defined therapeutics. Polym. Chem..

[12] Hatakeyama H., Akita H., Harashima H. (2013). The polyethyleneglycol dilemma: advantage and disadvantage of PEGylation of liposomes for systemic genes and nucleic acids delivery to tumors. Biol. Pharm. Bull..

[13] Amoozgar Z., Yeo Y. (2012). Recent advances in stealth coating of nanoparticle drug delivery systems. Wiley Interdiscip. Rev.: Nanomed. Nanobiotechnol..

[14] Zhang Z., Chen S., Jiang S. (2006). Dual-Functional biomimetic materials: nonfouling poly(carboxybetaine) with active functional groups for protein immobilization. Biomacromolecules.

[15] Choi H.S., Liu W., Liu F., Nasr K., Misra P., Bawendi M.G., Frangioni J.V. (2010). Design considerations for tumour-targeted nanoparticles. Nat. Nanotechnol..

[16] Blackman L.D., Gunatillake P.A., Cass P., Locock K.E.S. (2019). An introduction to zwitterionic polymer behavior and applications in solution and at surfaces. Chem. Soc. Rev..

[17] Laschewsky A. (2014). Structures and synthesis of zwitterionic polymers. Polymers.

[18] Zhou B., Li J., Lu B., Wu W., Zhang L., Liang J., Yi J., Li X. (2020). Novel polyzwitterion shell with adaptable surface chemistry engineered to enhance anti-fouling and intracellular imaging of detonation nanodiamonds under tumor pHe. Front. Mater. Sci..

[19] Fujii S., Takano S., Nakazawa K., Sakurai K. (2022). Impact of zwitterionic polymers on the tumor permeability of molecular bottlebrush-based nanoparticles. Biomacromolecules.

[20] Fujii S., Sakurai K. (2022). Zwitterionic amino acid polymer-grafted core-crosslinked particle toward tumor delivery. Biomacromolecules.

[21] Li Y., Yang H.Y., Thambi T., Park J.-H., Lee D.S. (2019). Charge-convertible polymers for improved tumor targeting and enhanced therapy. Biomaterials.

[22] Liu N., Han J., Zhang X., Yang Y., Liu Y., Wang Y., Wu G. (2016). pH-responsive zwitterionic polypeptide as a platform for anti-tumor drug delivery. Colloids Surf., B.

[23] Theodorou I., Anilkumar P., Lelandais B., Clarisse D., Doerflinger A., Gravel E., Ducongé F., Doris E. (2015). Stable and compact zwitterionic polydiacetylene micelles with tumor-targeting properties. Chem. Commun..

[24] Leiske M.N., Kempe K. (2022). A guideline for the synthesis of amino acid-functionalized monomers and their polymerizations. Macromol. Rapid Commun..

[25] Takano S., Sakurai K., Fujii S. (2021). Internalization into cancer cells of zwitterionic amino acid polymers via amino acid transporter recognition. Polym. Chem..

[26] Leiske M.N., Mazrad Z.A.I., Zelcak A., Wahi K., Davis T.P., McCarroll J.A., Holst J., Kempe K. (2022). Zwitterionic amino acid-derived polyacrylates as smart materials exhibiting cellular specificity and therapeutic activity. Biomacromolecules.

[27] Matsuura M., Ohshima M., Hiruta Y., Nishimura T., Nagase K., Kanazawa H. (2018). LAT1-targeting thermoresponsive fluorescent polymer probes for cancer cell imaging. Int. J. Mol. Sci..

[28] Carr L., Cheng G., Xue H., Jiang S. (2010). Engineering the polymer backbone to strengthen nonfouling sulfobetaine hydrogels. Langmuir.

[29] Leiske M.N., Lai M., Amarasena T., Davis T.P., Thurecht K.J., Kent S.J., Kempe K. (2021). Interactions of core cross-linked poly(2-oxazoline) and poly(2-oxazine) micelles with immune cells in human blood. Biomaterials.

[30] Morgese G., Verbraeken B., Ramakrishna S.N., Gombert Y., Cavalli E., Rosenboom J.-G., Zenobi-Wong M., Spencer N.D., Hoogenboom R., Benetti E.M. (2018). Chemical design of non-ionic polymer brushes as biointerfaces: poly(2-oxazine)s outperform both poly(2-oxazoline)s and PEG. Angew. Chem., Int. Ed..

[31] Krȩżel A., Bal W. (2004). A formula for correlating pKa values determined in D2O and H2O. J. Inorg. Biochem..

[32] Dinda P., Anas M., Banerjee P., Mandal T.K. (2022). Dual thermoresponsive boc-lysine-based acryl polymer: RAFT kinetics and anti-protein-fouling of its zwitterionic form. Macromolecules.

[33] Mahmoud A.M., Nowell C.J., Feeney O., van ’t Hag L., Davis T.P., Kempe K. (2022). Hydrophobicity regulates the cellular interaction of cyanine5-labeled poly(3-hydroxypropionate)-based comb polymers. Biomacromolecules.

[34] Yamada N., Honda Y., Takemoto H., Nomoto T., Matsui M., Tomoda K., Konno M., Ishii H., Mori M., Nishiyama N. (2017). Engineering tumour cell-binding synthetic polymers with sensing dense transporters associated with aberrant glutamine metabolism. Sci. Rep..

[35] Leiske M.N., Walker J.A., Zia A., Fletcher N.L., Thurecht K.J., Davis T.P., Kempe K. (2020). Synthesis of biscarboxylic acid functionalised EDTA mimicking polymers and their ability to form Zr(iv) chelation mediated nanostructures. Polym. Chem..

[36] Trommsdorff V.E., Köhle H., Lagally P. (1948). Zur polymerisation des methacrylsäuremethylesters1. Makromol. Chem..

[37] Perrier S. (2017). 50th anniversary perspective: RAFT polymerization—a user guide. Macromolecules.

[38] Grubisic Z., Rempp P., Benoit H. (1967). A universal calibration for gel permeation chromatography. J. Polym. Sci. B Polym. Lett..

[39] Jovic K., Nitsche T., Lang C., Blinco J.P., De Bruycker K., Barner-Kowollik C. (2019). Hyphenation of size-exclusion chromatography to mass spectrometry for precision polymer analysis – a tutorial review. Polym. Chem..

[40] Pogliani L. (1992). Molecular connectivity model for determination of isoelectric point of amino acids. J. Pharm. Sci..

[41] Buwalda S.J., Dijkstra P.J., Calucci L., Forte C., Feijen J. (2010). Influence of amide versus ester linkages on the properties of eight-armed PEG-PLA star block copolymer hydrogels. Biomacromolecules.

[42] Zhang X.N., Du C., Wang Y.J., Hou L.X., Du M., Zheng Q., Wu Z.L. (2022). Influence of the α-methyl group on elastic-to-glassy transition of supramolecular hydrogels with hydrogen-bond associations. Macromolecules.

[43] Fane A.G., Fell C.J.D., Suki A. (1983). The effect of ph and ionic environment on the ultrafiltration of protein solutions with retentive membranes. J. Membr. Sci..

[44] Khoerunnisa, I I., Mazrad Z.A., Park S.Y. (2017). pH-switchable bacteria detection using zwitterionic fluorescent polymer. Biosens. Bioelectron..

[45] Erfani A., Seaberg J., Aichele C.P., Ramsey J.D. (2020). Interactions between biomolecules and zwitterionic moieties: a review. Biomacromolecules.

[46] Kim Y., Binauld S., Stenzel M.H. (2012). Zwitterionic guanidine-based oligomers mimicking cell-penetrating peptides as a nontoxic alternative to cationic polymers to enhance the cellular uptake of micelles. Biomacromolecules.

[47] Cai S., Alhowyan A.A.B., Yang Q., Forrest W.C.M., Shnayder Y., Forrest M.L. (2014). Cellular uptake and internalization of hyaluronan-based doxorubicin and cisplatin conjugates. J. Drug Target..

[48] Altman B.J., Stine Z.E., Dang C.V. (2016). From Krebs to clinic: glutamine metabolism to cancer therapy. Nat. Rev. Cancer.

[49] Cha Y.J., Kim E.-S., Koo J.S. (2018). Amino acid transporters and glutamine metabolism in breast cancer. Int. J. Mol. Sci..

[50] Mahmoud A.M., de Jongh P.A.J.M., Briere S., Chen M., Nowell C.J., Johnston A.P.R., Davis T.P., Haddleton D.M., Kempe K. (2019). Carboxylated cy5-labeled comb polymers passively diffuse the cell membrane and target mitochondria. ACS Appl. Mater. Interfaces.

[51] Jiang Z., Liu H., He H., Yadava N., Chambers J.J., Thayumanavan S. (2020). Anionic polymers promote mitochondrial targeting of delocalized lipophilic cations. Bioconjugate Chem..

[52] Schöttler S., Becker G., Winzen S., Steinbach T., Mohr K., Landfester K., Mailänder V., Wurm F.R. (2016). Protein adsorption is required for stealth effect of poly(ethylene glycol)- and poly(phosphoester)-coated nanocarriers. Nat. Nanotechnol..

[53] Rajan R., Hayashi F., Nagashima T., Matsumura K. (2016). Toward a molecular understanding of the mechanism of cryopreservation by polyampholytes: cell membrane interactions and hydrophobicity. Biomacromolecules.

[54] Liu Z., Zhang Z., Zhou C., Jiao Y. (2010). Hydrophobic modifications of cationic polymers for gene delivery. Prog. Polym. Sci..

[55] Ramamurthi P., Zhao Z., Burke E., Steinmetz N.F., Müllner M. (2022). Tuning the hydrophilic–hydrophobic balance of molecular polymer bottlebrushes enhances their tumor homing properties. Adv. Healthc. Mater..

[56] Hadidi M., Zydney A.L. (2014). Fouling behavior of zwitterionic membranes: impact of electrostatic and hydrophobic interactions. J. Membr. Sci..

[57] Hühn D., Kantner K., Geidel C., Brandholt S., De Cock I., Soenen S.J.H., Rivera_Gil P., Montenegro J.-M., Braeckmans K., Müllen K., Nienhaus G.U., Klapper M., Parak W.J. (2013). Polymer-Coated nanoparticles interacting with proteins and cells: focusing on the sign of the net charge. ACS Nano.

[58] Algarni A., Pilkington E.H., Suys E.J.A., Al-Wassiti H., Pouton C.W., Truong N.P. (2022). In vivo delivery of plasmid DNA by lipid nanoparticles: the influence of ionizable cationic lipids on organ-selective gene expression. Biomater. Sci..

[59] Vu M.N., Kelly H.G., Wheatley A.K., Peng S., Pilkington E.H., Veldhuis N.A., Davis T.P., Kent S.J., Truong N.P. (2020). Cellular interactions of liposomes and PISA nanoparticles during human blood flow in a microvascular network. Small.

[60] van Geldermalsen M., Quek L.-E., Turner N., Freidman N., Pang A., Guan Y.F., Krycer J.R., Ryan R., Wang Q., Holst J. (2018). Benzylserine inhibits breast cancer cell growth by disrupting intracellular amino acid homeostasis and triggering amino acid response pathways. BMC Cancer.

[61] Wang Q., Beaumont K.A., Otte N.J., Font J., Bailey C.G., Geldermalsen M., Sharp D.M., Tiffen J.C., Ryan R.M., Jormakka M., Haass N.K., Rasko J.E.J., Holst J. (2014). Targeting glutamine transport to suppress melanoma cell growth. Int. J. Cancer.

[62] Grewer C., Grabsch E. (2004). New inhibitors for the neutral amino acid transporter ASCT2 reveal its Na+-dependent anion leak. J. Physiol..

[63] Ramachandran S., Sennoune S.R., Sharma M., Thangaraju M., Suresh V.V., Sneigowski T., Bhutia Y.D., Pruitt K., Ganapathy V. (2021). Expression and function of SLC38A5, an amino acid-coupled Na+/H+ exchanger, in triple-negative breast cancer and its relevance to macropinocytosis. Biochem. J..

[64] Nanga R.P., DeBrosse C., Singh A., D'Aquilla K., Hariharan H., Reddy R. (2014). Glutaminase catalyzes reaction of glutamate to GABA. Biochem. Biophys. Res. Commun..

[65] Bannai S. (1986). Exchange of cystine and glutamate across plasma membrane of human fibroblasts. J. Biol. Chem..

[66] Sharma M.K., Seidlitz E.P., Singh G. (2010). Cancer cells release glutamate via the cystine/glutamate antiporter. Biochem. Biophys. Res. Commun..

[67] Abramson H.A., Moyer Electrokinetic Phenomena L.S. (1938). XIII. A comparison of the isoelectric points of dissolved and crystalline amino acids. J. Gen. Physiol..

[68] Gout P.W., Buckley A.R., Simms C.R., Bruchovsky N. (2001). Sulfasalazine, a potent suppressor of lymphoma growth by inhibition of the xc− cystine transporter: a new action for an old drug. Leukemia.

[69] Scalise M., Galluccio M., Console L., Pochini L., Indiveri C. (2018). The human SLC7A5 (LAT1): the intriguing histidine/large neutral amino acid transporter and its relevance to human health. Front. Chem..

[70] Scalise M., Pochini L., Console L., Losso M.A., Indiveri C. (2018). The human SLC1A5 (ASCT2) amino acid transporter: from function to structure and role in cell Biology. Front. Cell Dev. Biol..

[71] Sloan J.L., Mager S. (1999). Cloning and functional expression of a human Na+ and Cl−-dependent neutral and cationic amino acid transporter B0+. J. Biol. Chem..

