# Supplementary material for: Impact of the polymer backbone chemistry on interactions of amino-acid-derived zwitterionic polymers with cells
Source: Bioact Mater. 2023 Jan 10;24:524–34. doi: 10.1016/j.bioactmat.2023.01.005 (PMC9860433; doi:10.1016/j.bioactmat.2023.01.005)
Supplement: Multimedia component 1 [file mmc1.pdf]

Supporting information

Impact of the polymer backbone chemistry on interactions of amino-acid-derived zwitterionic polymers with cells

Meike N. Leiske,[a,#,] Bruno De Geest,[b] Richard Hoogenboom[a,*]*
[a]Supramolecular Chemistry Group, Centre of Macromolecular Chemistry (CMaC), Department of Organic and Macromolecular Chemistry, Ghent University, Krijgslaan 281 S4, B-9000 Ghent, Belgium
[b]Department of Pharmaceutics and Cancer Research Institute Ghent (CRIG), Ghent University, Ottergemsesteenweg 460, B-9000 Ghent, Belgium
[#]Current address: Faculty of Biology, Chemistry & Earth Sciences, University of Bayreuth, Universitätsstraße 30, 95447 Bayreuth, Germany
*meike.leiske@uni-bayreuth.de; richard.hoogenboom@ugent.be

Experimental part

Materials and instrumentation

Materials. Basic alumina (Carl Roth), lauroyl peroxide (Luperox, Acros Organics), toluene (Sigma-Aldrich), ethyl acetate (Sigma-Aldrich), *n*-hexane (Sigma-Aldrich), 2-hydroxyethyl acrylate (HEA, TCI), 2-hydroxyethyl acrylamide (HEAAm, TCI), 2-hydroxyethyl methacrylate (HEMA, TCI), 2-isopropenyl-2-oxazoline (iPOx, Sigma-Aldrich), *L*-glutamic acid (L-Glu, Sigma-Aldrich), *L*-phenylalanine (L-Phe, Sigma-Aldrich), *L*-leucine (L-Leu, Sigma-Aldrich), *L*-glutamine (L-Gln, Gibco, 200 mM solution) Boc-*L*-glutamic acid *α*-tert-butyl ester (Sigma Aldrich), *L*-serine-*O*-benzyl ether (BzlSer, Sigma Aldrich), anhydrous dichloromethane (DCM, Sigma-Aldrich), anhydrous *N*,*N*-dimethylformamide (DMF, Sigma-Aldrich), N,N-dimethylacetamide (DMAc, HPLC grade, 99.8+%), lithium chloride (LiCl, Fisher Scientific), anhydrous dimethylsulfoxide (DMSO, Sigma-Aldrich), *N*,*N'*-dicyclohexylcarbodiimide (DCC, Sigma-Aldrich), *N*,*N*'-diisopropylcarbodiimide (DIC, Sigma-Aldrich) and 4-dimethylaminopyridine (DMAP, Sigma Aldrich), 6-aminofluorescein (6AF, TCI), 4-cyano-4-[(dodecylsulfanylthiocarbonyl)sulfanyl]pentanoic acid (CDTA, Sigma-Aldrich), Dulbecco’s phosphate buffered saline (DPBS, Gibco), fetal bovine serum (FBS, Gibco), Dulbecco's Modified Eagle Medium: Nutrient Mixture F-12 (DMEM/F12, Gibco), Trypsin (Gibco), were used as received.
Deionised water was prepared with a resistivity less than 18.2 MΩ cm using an Arium 611 from Sartorius with the Sartopore 2 150 (0.45 + 0.2 µm pore size) cartridge filter.
Azobisisobutyronitrile (AIBN, Sigma-Aldrich) was recrystallised from methanol prior to use. All other chemicals were purchased from standard suppliers and used as received.

^{1}H and ^{13}C Distorsionless Enhancement by Polarization Transfer (DEPT) Nuclear Magnetic Resonance (NMR) spectroscopy. NMR spectroscopy of all samples was carried out using a Bruker AVANCE III HD 300 MHz or 400 MHz spectrometer as indicated utilising deuterated solvents obtained from Sigma-Aldrich.

Size-exclusion chromatography (SEC). SEC was performed on two different systems. For protected polymers and POEGMA, an Agilent 1260-series HPLC system equipped with a 1260 online degasser, a 1260 ISO-pump, a 1260 automatic liquid sampler, a thermostatted column compartment at 50 °C equipped with two PLgel 5 µm mixed-D columns and a mixed-D guard column in series, a 1260 diode array detector, and a 1260 refractive index detector. The used eluent was DMAc containing 50 mm of LiCl at a flow rate of 0.500 mL min^{-1}. The spectra were analysed using the Agilent ChemStation software with the SEC add on. Molar mass and dispersity (*Đ*) values were determined by SEC-analysis, calculated against poly(methylmethacrylate) (PMMA) standards. SEC of deprotected polymers was performed on an Agilent 1260-series HPLC system equipped with an online PSS degasser, a 1260 ISO-pump, a 1260 automatic liquid sampler (ALS), a 1261 thermostatted column compartment (TCC) at 30 °C equipped with two PSS Novema Max 5 µm columns and a precolumn in series, a 1262 diode array detector (DAD) and a 1290 refractive index detector (RID). The used eluent was an acetate buffer at pH 3.6 containing 30% acetonitrile and 0.1M $NaNO_3$ at a flow rate of 0.500 ml/min. The spectra were analysed using the Agilent Chemstation software with the GPC add on. Molar mass values and Đ values were calculated against PEG standards from PSS. Light scattering (LS) measurements were performed on a 3-angle static light scattering (MALS) detector, *i.e.* miniDAWN TREOS, from Wyatt Technology. The detector was coupled on-line to an Agilent 1260 infitiny HPLC system (*vide* DMA-SEC) and used to determine absolute molar mass of the analysed polymer samples. The measurements were performed at ambient temperature, *i.e.* no temperature control unit is supplied/installed with the above mentioned LS detector. The refractive index (RI) increment (dn/dc) values were either used as reported for the certain polymer in DMAc or determined *via* online SEC equipped with an RI detector, which measured the RI increase for a 1 to 10 mg mL^{-1} concentration series of the mentioned polymers. The LS results were further analysed with the provided Astra 7 software, also designed by Wyatt Technology.

Fluorescence. The fluorescence measurements were carried out on a Cary Eclipse fluorescence spectrophotometer equipped with a Varian Cary Temperature Controller. The emission spectra resulting from excitation by a 488 nm laser with photomultiplier tube voltage at 400 V were monitored from 495 to 600 nm, and the slit width of the excitation and emission were kept at 5 nm during the measurements.

Lyophilisation. Lyophilisation of samples was conducted using an Alpha 1−2 LDplus freeze-dryer from Martin Christ Gefriertrocknungsanlagen GmbH (Germany).

Dynamic light scattering (DLS). DLS was measured on a Zetasizer Nano-ZS Malvern apparatus (Malvern Instruments Ltd) using disposable cuvettes. The excitation light source was a He–Ne laser at 633 nm and the intensity of the scattered light was measured at an angle of 173°. This method measures the rate of intensity fluctuation, and the size of the particles is determined through the Stokes–Einstein equation. The concentration of the polymer solution was 1 mg mL^{-1} (in diH_2O, DPBS or DMEM/F12) in all cases.

Electrophoretic light scattering (ELS). ELS was used to measure the zeta potential (ζ). The measurement was also performed on the Zetasizer Nano ZS by applying laser Doppler velocimetry. For each measurement, 20 runs were carried out using the slow-field reversal and the fast-field reversal mode at 150 V. Each experiment was performed in triplicates at 25 °C. The zeta potential was calculated from the electrophoretic mobility (μ) according to the Henry equation. Henry coefficient f(ka) was calculated according to Ohshima.[1]

Mass spectrometry. ESI-MS spectra were acquired on a quadrupole ion trap LC mass spectrometer (Thermo Finnigan MAT LCQ mass spectrometer) equipped with electrospray ionization.

Synthesis and characterisation

Synthesis of *N*Boc-Glu-O*t*Bu-acrylate (*N*Boc-Glu-O*t*Bu-A), *N*Boc-Glu-O*t*Bu-methacrylate (*N*Boc-Glu-O*t*Bu-MA), and *N*Boc-Glu-O*t*Bu-acrylamide (*N*Boc-Glu-O*t*Bu-AAm). The synthesis of amino acid containing acrylates was conducted according to a literature procedure[2] and is described exemplarily for *N*Boc-Glu-O*t*Bu-A.

In a single-neck round-bottom flask 5.0 g *N*Boc-Glu-O*t*Bu (16.5 mmol, 1.0 equiv.) were dissolved in 50 mL anhydrous DCM and the solution was purged with Ar for 30 min. While purging with Ar, 201 mg DMAP (1.65 mmol, 0.1 equiv.), and 3.74 g DCC (18.1 mmol, 1.1 equiv.) were added. Subsequently, the reaction mixture was cooled in an ice bath. While stirring, 2.11 g HEA (18.1 mmol, 1.1 equiv.) were added dropwise. The reaction mixture was stirred at 0 °C for another 30 min before warming up to room temperature and stirring overnight. Following the reaction, the newly formed *N*,*N'*-dicyclohexylurea was removed from the crude product by suction filtration. After that, deionized water was added and extracted with DCM (3×). The combined organic phases were washed with a saturated $NaHCO_3$ solution (3×) and brine (1×). After drying of the organic layer over Na_2SO_4, the salt was filtered off and the solvent was removed under reduced pressure using a rotary evaporator. Column chromatography using silica gel as the solid phase and a solvent mixture (gradient of n-hexane/EtOAc (4:1) to 100% EtOAc) as eluent was

used to further purify the crude product. After removal of the solvent under reduced pressure and complete drying *in vacuo*, the product was obtained as a colourless viscous liquid.

*N*Boc-Glu-O*t*Bu-A. Yield: 3.9 g, 59%.

^{1}H NMR (400 MHz) in $CDCl_3$: δ = 6.37 (1H, d, C*H*-CH-), 6.08 (1H, dd, CH_2-C*H*-C=O-), 5.80 (1H, d, C*H*-CH-), 5.02 (1H, s, -CH-N*H*-CO-), 4.28 (4H, m, -O-CH_2-CH_2-O-), 4.07 (1H, m, -CH2-C*H*-NH-), 1.81 – 2.44 (4H, m, -C=O-CH_2-CH_2-CH-NH-), 1.40 (9H, s, $(CH_3)_3$-C-), 1.37 (9H, s, $(CH_3)_3$-C-) ppm.

*N*Boc-Glu-O*t*Bu-MA. Yield: 3.1 g, 45%.

^{1}H NMR (400 MHz) in $CDCl_3$: δ = 6.07 (1H, m, C*H*-C-C=O-), 5.52 (1H, m, C*H*-C-C=O-), 5.01 (1H, s, -CH-N*H*-CO-), 4.28 (4H, m, -O-CH_2-CH_2-O-), 4.14 (1H, m, -CH_2-C*H*-NH-), 1.88 (3H, m, CH_3-C-C=O-), 1.79 – 2.43 (4H, m, -C=O-CH_2-CH_2-CH-NH-), 1.40 (9H, s, $(CH_3)_3$-C-), 1.38 (9H, s, $(CH_3)_3$-C-) ppm.

^{13}C NMR (100 MHz) in $CDCl_3$: δ = 172.6 (-O-*C*O-CH_2-), 171.5 (-CH-*C*O-O-), 167.2 (-C-*C*O-O-), 155.4 (-NH-*C*O-O-), 136.0 (CH_2=*C*-CO-), 126.1 (*C*H_2=C-CO-), 82.2 (-O-*C*-$(CH_3)_3$ (Boc)), 80.0 (-O-*C*-$(CH_3)_3$ (*t*Bu)), 62.4 (-O-*C*H_2-*C*H_2-O-), 53.4 (-CH_2-*C*H-NH-), 30.3, 28.4, 28.0, 18.3 ppm.

HR-ESI MS: calcd for $[C_{20}H_{33}NO_8 + Na]^+$ m/z = 438.2104 Da; found m/z = 438.2097 Da

*N*Boc-Glu-O*t*Bu-AAm. Yield: 2.6 g, 39%.

^{1}H NMR (400 MHz) in $CDCl_3$: δ = 6.87 (1H, s, -O=C-N*H*-CH_2-), 6.25 (1H, dd, C*H*-CH-), 6.13 (1H, dd, CH_2-C*H*-C=O-), 5.55 (1H,dd, C*H*-CH-), 5.09 (1H, d *br*, -CH-N*H*-CO-), 3.99 – 4.39 (3H, m, -CH_2-CH_2-O- and -CH_2-C*H*-NH-), 3.40 – 3.72 (2H, m, -NH-CH_2-CH_2-), 1.68 – 2.45 (4H, m, -C=O-CH_2-CH_2-CH-NH-), 1.41 (9H, s, $(CH_3)_3$-C-), 1.38 (9H, s, $(CH_3)_3$-C-) ppm.

^{13}C NMR (100 MHz) in $CDCl_3$: δ = 130.9 (CH_2-*C*H-CO-), 126.4 (*C*H_2-CH-CO-), 82.6 (-CH_2-*C*H_2-O-), 63.7 (-NH-*C*H_2-CH_2-), 52.9 (-CH_2-*C*H-NH-), 38.7 (-CO-*C*H_2-CH_2-), 30.2 (-CH_2-*C*H_2-CH-), 28.4 (-(*C*H_3)$_2$-C-), 28.0 (-(*C*H_3)$_2$-C-) ppm.

HR ESI-MS: calcd for $[C_{19}H_{32}N_2O_7 + Na]^+$ m/z = 423.2107 Da; found m/z = 423.2087 Da

Synthesis of *N*Boc-Glu-O*t*Bu-methacrylamide (*N*Boc-Glu-O*t*Bu-MAAm). The synthesis of *N*Boc-Glu-O*t*Bu-MAAm was modified from a literature procedure that described the synthesis of methacylamide monomers using iPOx.[3] In a 100 mL round bottom flask, 5.0 g *N*Boc-Glu-O*t*Bu (16.5 mmol, 1.0 equiv.) and 1.5 g iPOx (13.2 mmol, 0.8 equiv.) were dissolved in 40 mL anhydrous DMF. Subsequently the flask was sealed with a rubber septum and heated to 136 °C with a heating block. After stirring at 136 °C for 24 h, the reaction mixture was cooled to RT and precipitated into an excess of diH_2O. After centrifugation (10,000 rpm, 10 min) the supernatant was discarded, and the crude product was lyophilised to remove any solvent traces. After that, 200 mL of chloroform were added to redissolve the product and it was washed with sat. aq. $NaHCO_3$ (3 × 100 mL) and brine (1 × 100

mL). The organic layer was dried over Na_2SO_4. Then, the salt was filtered off and the solvent was removed under reduced pressure. Column chromatography using silica gel as the solid phase and a solvent mixture (gradient of n-hexane/EtOAc (4:1) to 100% EtOAc) as eluent was used to further purify the crude product. After removal of the solvent under reduced pressure and complete drying in vacuo, the product was obtained as a white solid.

Yield: 2.93 g, 43%.

^{1}H NMR (100 MHz) in $CDCl_3$: δ = 6.60 (1H, s, -O=C-N*H*-CH_2-), 5.64 (1H, s *br*, C*H*-C-C=O-), 5.26 (1H, s *br*, C*H*-C-C=O-), 5.04 (1H, d *br*, -CH-N*H*-CO-), 3.95 – 4.39 (3H, m, -CH_2-C*H₂*-O- and -CH2-C*H*-NH-), 3.35 – 3.68 (2H, m, -NH-C*H₂*-CH_2-), 1.90 (3H, m, C*H₃*-C-C=O-), 1.67 – 2.54 (4H, m, -C=O-C*H₂*-C*H₂*-CH-NH-), 1.40 (9H, s, (C*H₃*)₃-C-), 1.37 (9H, s, (C*H₃*)₃-C-) ppm.

^{13}C NMR (400 MHz) in $CDCl_3$: δ = 119.8 (*C*H_2-C-C=O-), 82.5 (-CH_2-*C*H_2-O-), 63.8 (-NH-*C*H_2-CH_2-), 53.1 (-CH_2-*C*H-NH-), 39.4 (-C=O-*C*H_2-CH_2-), 30.4 (*C*H_2-CH_2-CH-), 28.3 (-(*C*H_3)$_2$-C-) 28.0 (-(*C*H_3)$_2$-C-), 18.9 (*C*H_3-C-) ppm.

HR-ESI MS: calcd for $[C_{20}H_{34}N_2O_7 + Na]^+$ *m/z* = 437.2264 Da; found *m/z* = 437.2259 Da

Synthesis of 6AF-modified chain-transfer agent (CTA). In a reaction vial, 100 mg CDTA (0.25 mmol, 1.0 equiv.) were dissolved in 4 mL anhydrous DMF and the solution was purged with Ar. Subsequently, the reaction mixture was cooled in an ice bath. While purging with Ar, 6.0 mg DMAP (5×10^{-2} mmol, 0.1 equiv.), and 38 mg DIC (0.3 mmol, 1.1 equiv.) were added. In a separate vial, 95 mg 6-AF (0.27 mmol, 1.1 equiv.) were added dropwise. The reaction mixture was stirred in the dark at 0 °C for another 30 min before warming up to room temperature and stirring overnight.

After that, the crude product was purified by preparative size-exclusion chromatography using Sephadex LH20 as the solid phase and MeOH as eluent. After removal of the solvent under reduced pressure and complete drying *in vacuo*, diH_2O was added to lyophilise the product and obtain it as a yellow solid.

Yield: 70 mg (39%)

^{1}H NMR (400 MHz) in DMSO-d_6: δ = 9.98 (1H, s br, O*H*-Ar-), 8.10 (1H, d, *Ar*), 7.96 (1H, s, *Ar*), 7.56 (1H, d, *Ar*), 6.06 – 6.79 (6H, m, *Ar*), 10.17 (1H, s br, -CO-N*H*-Ar-), 3.62 (2H, t, -S-C*H₂*-CH_2-), 2.27 (-S-CH_2-C*H₂*-), 1.85 (2H, t, -C*H₂*-CO-NH-) 1.24 (18H, m, CH_3-(C*H₂*)₉-CH_2-) 0.86 (3H, t, C*H₃*-CH_2-CH_2-) ppm.

13C NMR (100 MHz) in DMSO-d_6: δ = 102.1 – 159.3 (multiple peaks, *Ar*), 45.9 (CH_3-(*C*H_2)$_{11}$-), 29.3 (-C-*C*H_2-CH_2-), 23.5 (-S-C-*C*H_3), 22.2 (-CH2-*C*H_2-CO-), 9.0 (-(CH_2)$_{11}$-*C*H_3) ppm.

HR ESI MS: calcd for $[C_{39}H_{44}N_2O_6S_3 + Na]^+$ *m/z* = 733.2440 Da; found *m/z* = 733.2399 Da

Synthesis of polymers *via* RAFT polymerisation. Polymerisations are exemplarily described for *N*Boc-Glu-O*t*Bu-A yielding P(*N*Boc-Glu-O*t*Bu-A_{136})-CTA. In a reaction vessel, 564 mg *N*Boc-Glu-O*t*Bu-A (1.4×10^{-3} mol, 150.0 equiv.), 0.15 mg AIBN (9.4×10^{-7} mol, 0.1 equiv.), and 3.5 mg CDTA (9.4×10^{-6} mol, 1.0 equiv.) were dissolved in 1.0 mL toluene and sealed with a rubber septum. Polymerisations of *N*Boc-Glu-O*t*Bu-MAAm and those using a fluoresceine-modified CTA were conducted in DMSO instead. The reaction mixture was deoxygenated with Ar for 30 min and subsequently placed in a preheated heating block and stirred at 70 °C for 24 h. The reaction was terminated by cooling to RT and purging air in. A sample was taken to determine the conversion via ^1H NMR spectroscopy in $CDCl_3$. The crude polymer was diluted with DCM and precipitated in ice-cold *n*-hexane. After centrifugation (6000 rpm, 1 min), the supernatant was discarded, and the polymer was dried under reduced pressure to obtain the product as a yellow solid. Analytical results are summarised in Table S1.

Removal of the Z-group of the CTA. The end-group removal of trithiocarbonates was conducted according to a literature procedure[4] and is described exemplarily for P(*N*Boc-Glu-O*t*Bu-A_{136})-CTA, yielding P(*N*Boc-Glu-O*t*Bu-A_{136}).

In a reaction vessel equipped with a stirrer bar, 400 mg P(*N*Boc-Glu-O*t*Bu-A_{136})-CTA (7.0×10^{-6} mol, 1.0 equiv.), 24 mg AIBN (1.5×10^{-4} mol, 20.0 equiv.) and 6 mg Luperox® (1.5×10^{-5} mol, 2.0 equiv.) were dissolved in 1 mL toluene. The vessel was sealed with a rubber septum and the reaction mixture was deoxygenated with Ar for 30 min. Subsequently, the vial was placed in a preheated heating block and heated to 80 °C for 2.5 h under continuous stirring. After cooling to RT, 1 mL of DCM was added and the crude mixture was precipitated in 40 mL ice-cold n-hexane, centrifuged (6000 rpm, 1 min) and the supernatant was discarded. The precipitation procedure was repeated thrice. The remaining solvent was evaporated under reduced pressure to obtain the product as a white (or green) solid. Complete removal of the trithiocarbonate end-group was confirmed by SEC measurements in DMAc using a UV detector (λ = 310 nm), showing the disappearance of the polymer UV trace.

Analytical results are summarised in Table S1.

Acidic deprotection of polymers. Acidic deprotection of *N*Boc and O*t*Bu protected polymers was conducted independently from their molar mass. The deprotection process is exemplarily described for P(*N*Boc-Glu-O*t*Bu-A_{136}) yielding P(Glu-OH-A_{136}).

In a reaction vessel, 50 mg of polymer were dissolved in 1 mL trifluoroacetic acid, and the reaction was stirred at room temperature for 1 h. Dye-labelled polymers were stirred in the dark.

Subsequently, the reaction mixture was diluted with 4 mL MeOH and the polymer was precipitated in 40 mL ice-cold diethyl ether. Then, the suspension was centrifuged (6000 rpm, 1 min) and the supernatant was discarded. The polymer

was re-dissolved in diH_2O and freeze-dried to obtain the product as a white (or green) powder.

The success of the deprotection of the polymer was analysed by ^{1}H NMR in D_2O, showing the disappearance of the BOC, respectively *t*Bu, signal around δ = 1.4 ppm.

Analytical results are summarised in **Table S1**.

Results

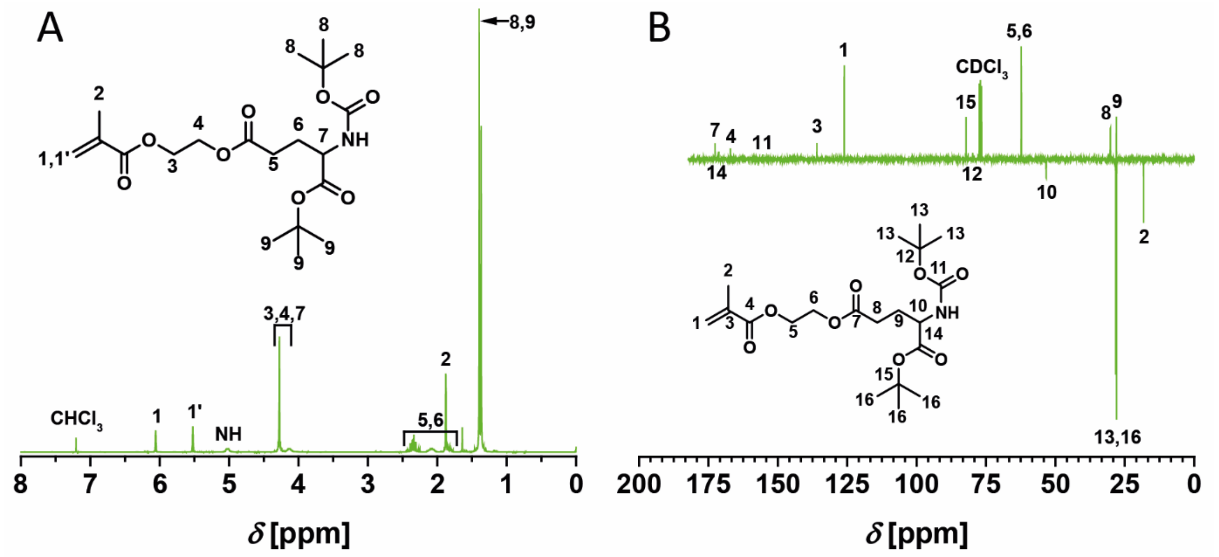

Figure S1. Characterisation of *N*Boc-Glu-O*t*Bu-MA. A: ^{1}H NMR spectrum (400 MHz, $CDCl_3$). B: ^{13}C (DEPT) NMR spectrum (100 MHz, $CDCl_3$).

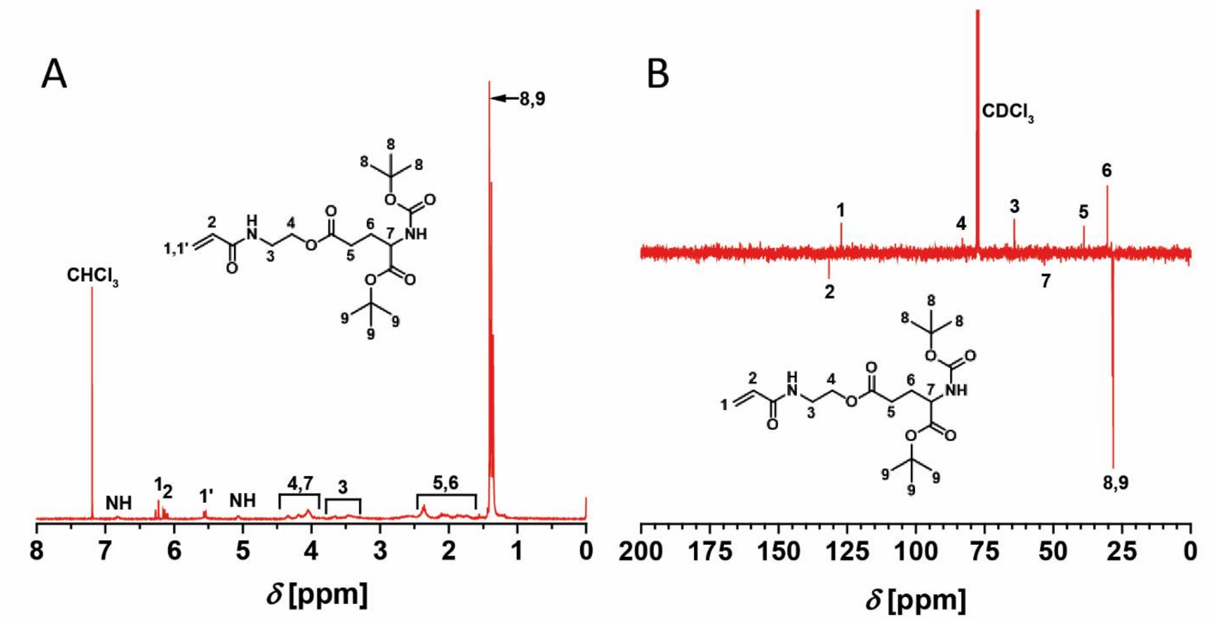

Figure S2. Characterisation of *N*Boc-Glu-O*t*Bu-AAm. A: ^{1}H NMR spectrum (400 MHz, $CDCl_3$). B: ^{13}C (DEPT) NMR spectrum (100 MHz, $CDCl_3$).

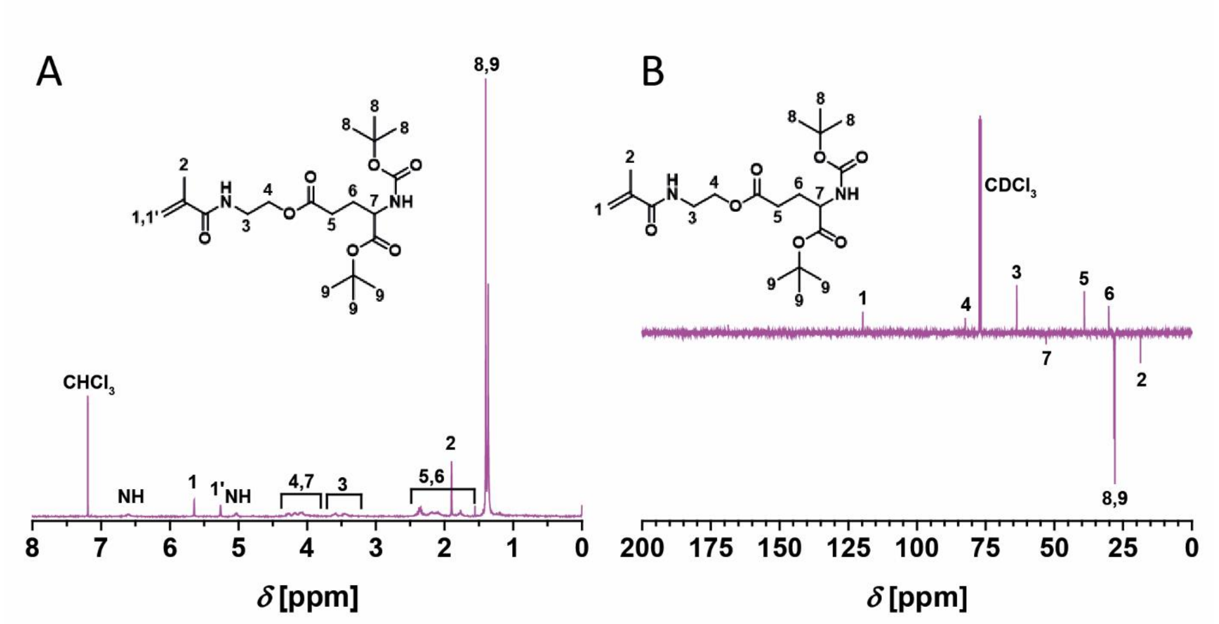

Figure S3. Characterisation of *N*Boc-Glu-O*t*Bu- MAAm. A: ^{1}H NMR spectrum (400 MHz, $CDCl_3$). B: ^{13}C NMR (DEPT) spectrum (100 MHz, $CDCl_3$).

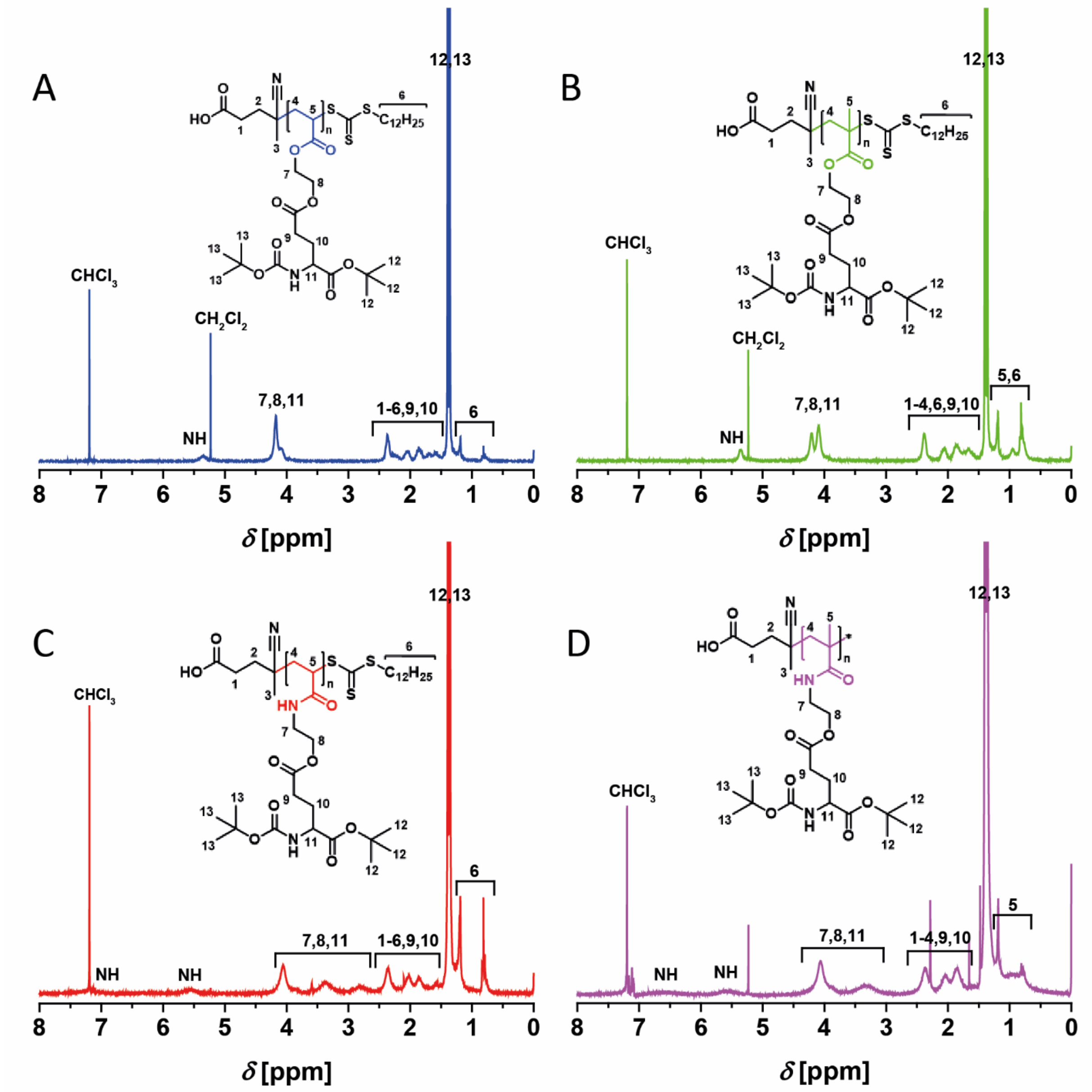

Figure S4. Characterisation of homopolymers after preparation via RAFT polymerisation by ^{1}H NMR spectroscopy (400 MHz) in $CDCl_3$ A: P(*N*Boc-Glu-O*t*Bu-A_{136})-CTA. B: P(*N*Boc-Glu-O*t*Bu-MA_{138})-CTA. C: P(*N*Boc-Glu-O*t*Bu-AAm_{140})-CTA. P(*N*Boc-Glu-O*t*Bu-$MAAm_{150}$)-CTA.

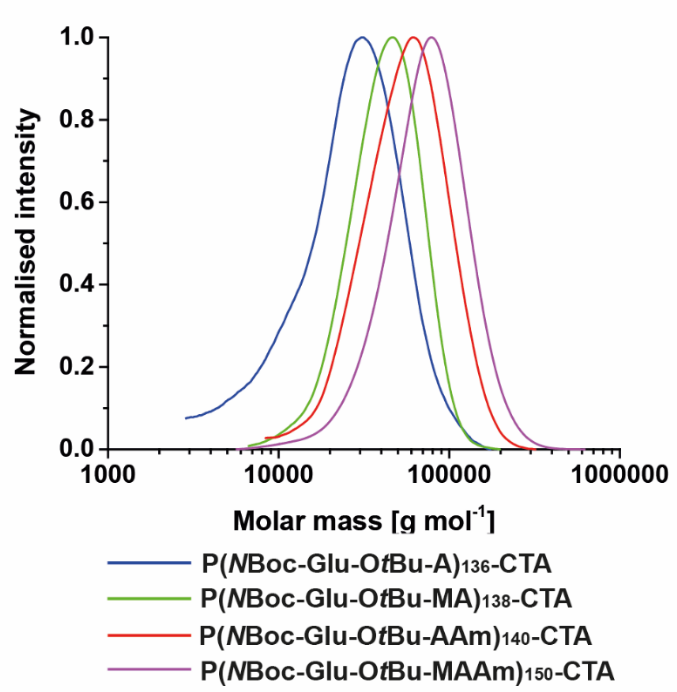

Figure S5. Characterisation of homopolymers after preparation via RAFT polymerisation by SEC measurements in DMAc. Molar mass distribution obtained from PMMA calibration. For elugram see Figure 1A. For SEC analysis after Z-group removal and acidic deprotection see Figure 1B, S7 and S9.

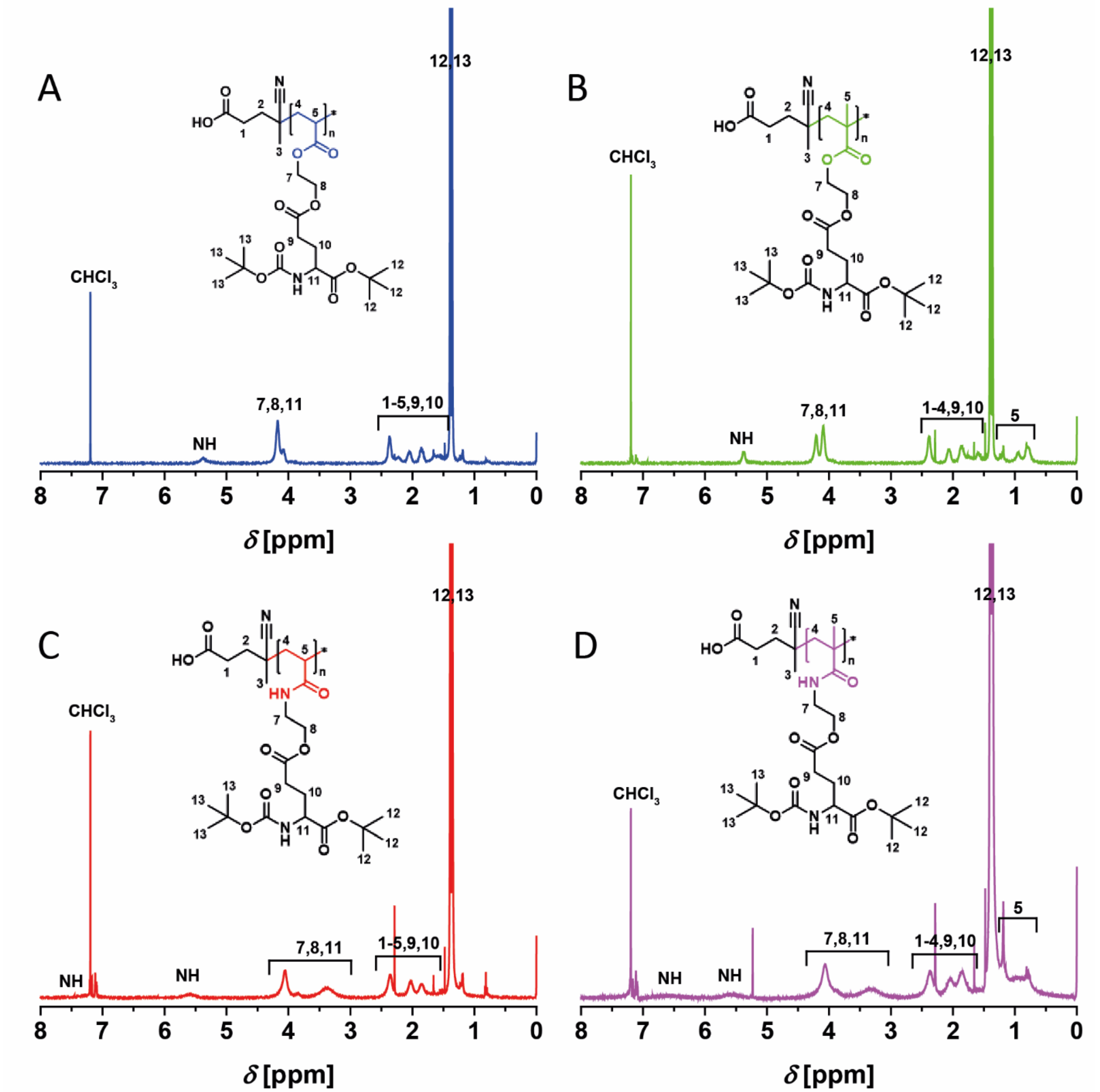

Figure S6. Characterisation of homopolymers after removal of the CTA Z-group by ^{1}H NMR spectroscopy (400 MHz) in $CDCl_3$ A: P(*N*Boc-Glu-O*t*Bu-A$_{136}$). B: P(*N*Boc-Glu-O*t*Bu-MA$_{138}$). C: P(*N*Boc-Glu-O*t*Bu-AAm$_{140}$). P(*N*Boc-Glu-O*t*Bu-MAAm$_{150}$).

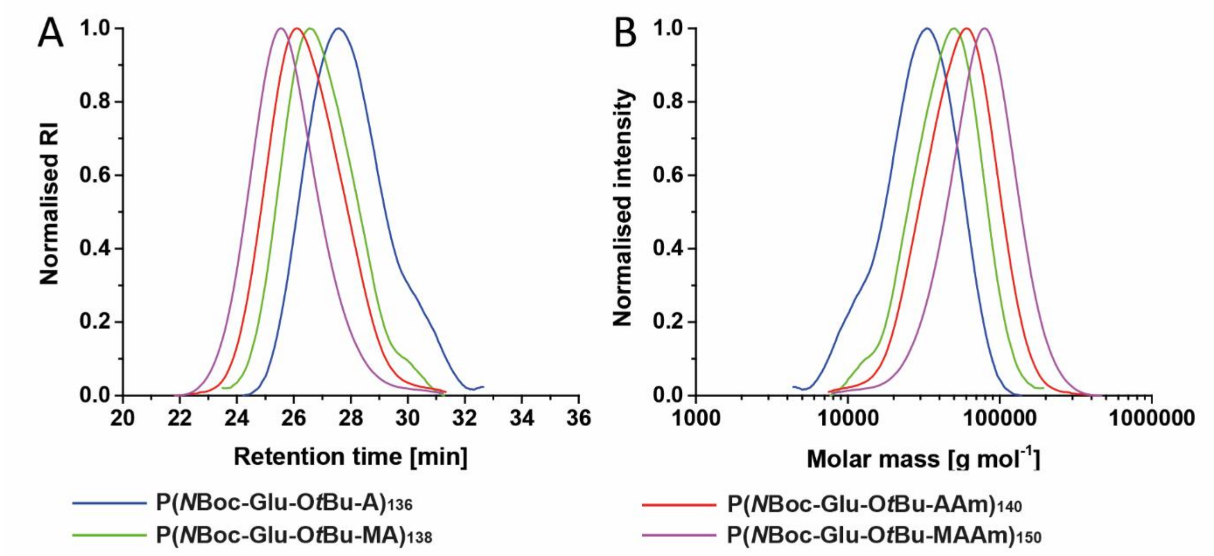

Figure S7. Characterisation of homopolymers after removal of the CTA Z-group by SEC measurements in DMAc. A: Elugram. B: Molar mass distribution obtained from PMMA calibration. For SEC analysis of polymers before Z-group removal see Figure 1A and S5. For SEC analysis of deprotected polymers see Figure 1B and S9.

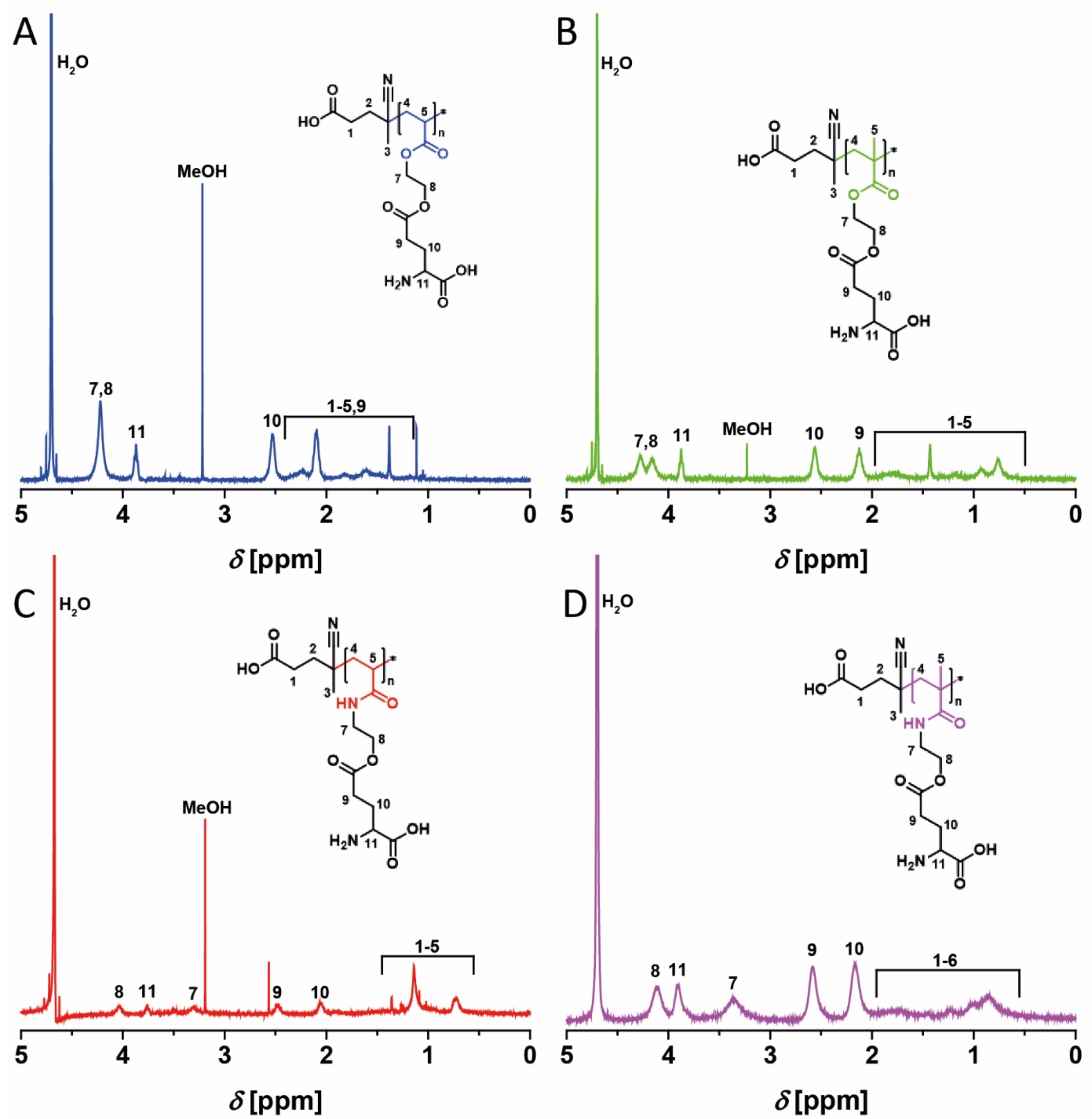

Figure S8. Characterisation of homopolymers after acidic deprotection by ^{1}H NMR spectroscopy (400 MHz) in D_2O A: P(Glu-OH-A_{136}). B: P(Glu-OH-MA_{138}). C: P(Glu-OH-AAm_{140}). P(Glu-OH-$MAAm_{150}$).

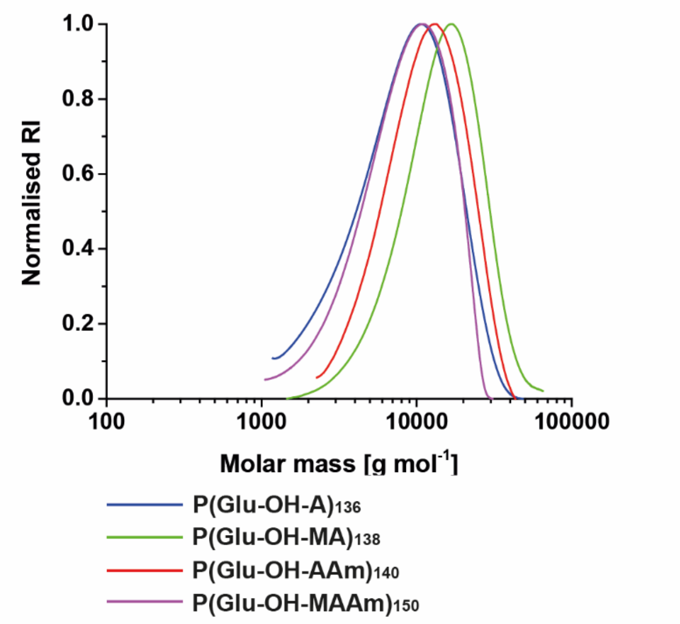

Figure S9. Characterisation of homopolymers after acidic deprotection by SEC measurements in acetate buffer at pH 3.6 containing 30% (v/v) MeCN and 0.1 M $NaNO_3$. Molar mass distribution obtained from PEG calibration. For elugram see Figure 1B. For SEC analysis of protected polymers before and after Z-group removal see Figure 1A, S5 and S7.

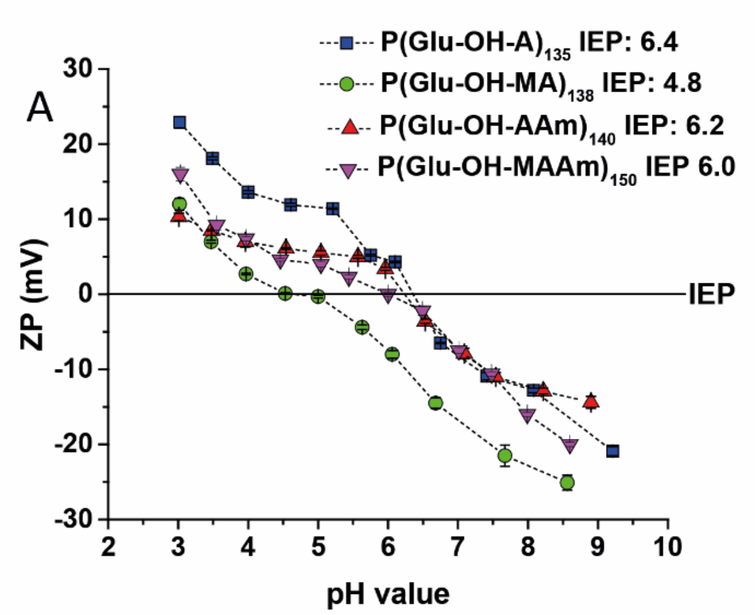

Figure S10. Zeta potential of different zwitterionic polymers determined by ELS measurements in dependence of the pH value. Polymers were dissolved in diH_2O at a concentration of 10 mg mL^{-1} and the pH value was adjusted by the addition of 0.01 M NaOH solution. The pH value at which the solution comprises zeta potential of 0 mV was defined as the isoelectric point (IEP). The dashed connecting line between the data points

is added to guide the eye. Scatter represents the mean and SD of 3 measurements of the same solution.

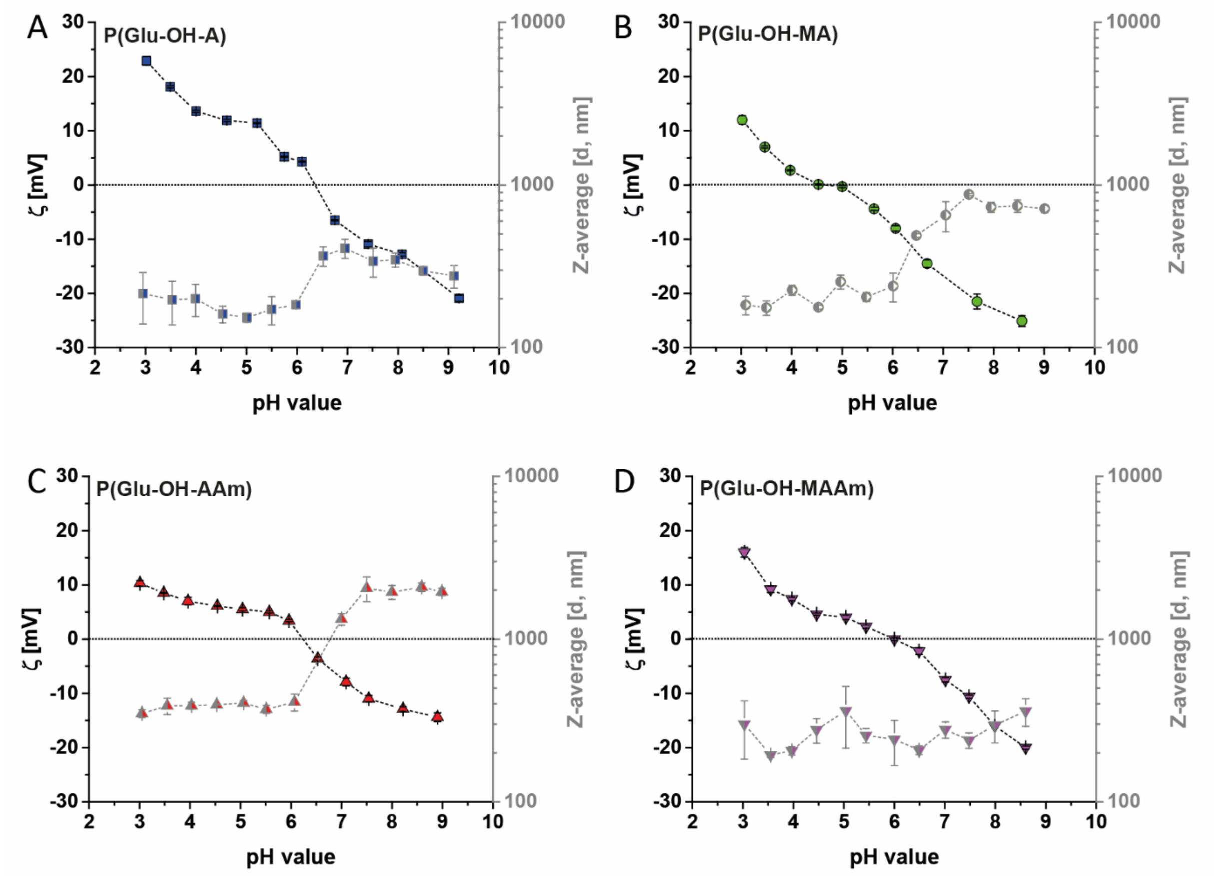

Figure S11. Zeta potential and size of different zwitterionic polymers determined by ELS and DLS measurements, respectively, in dependence of the pH value. Polymers were dissolved in diH_2O at a concentration of 10 mg mL^{-1} and the pH value was adjusted by the addition of 0.01 M NaOH solution. The dashed connecting line between the data points is added to guide the eye. Scatter represents the mean and SD of 3 measurements of the same solution. Dotted line indicates the isoelectric point (IEP).

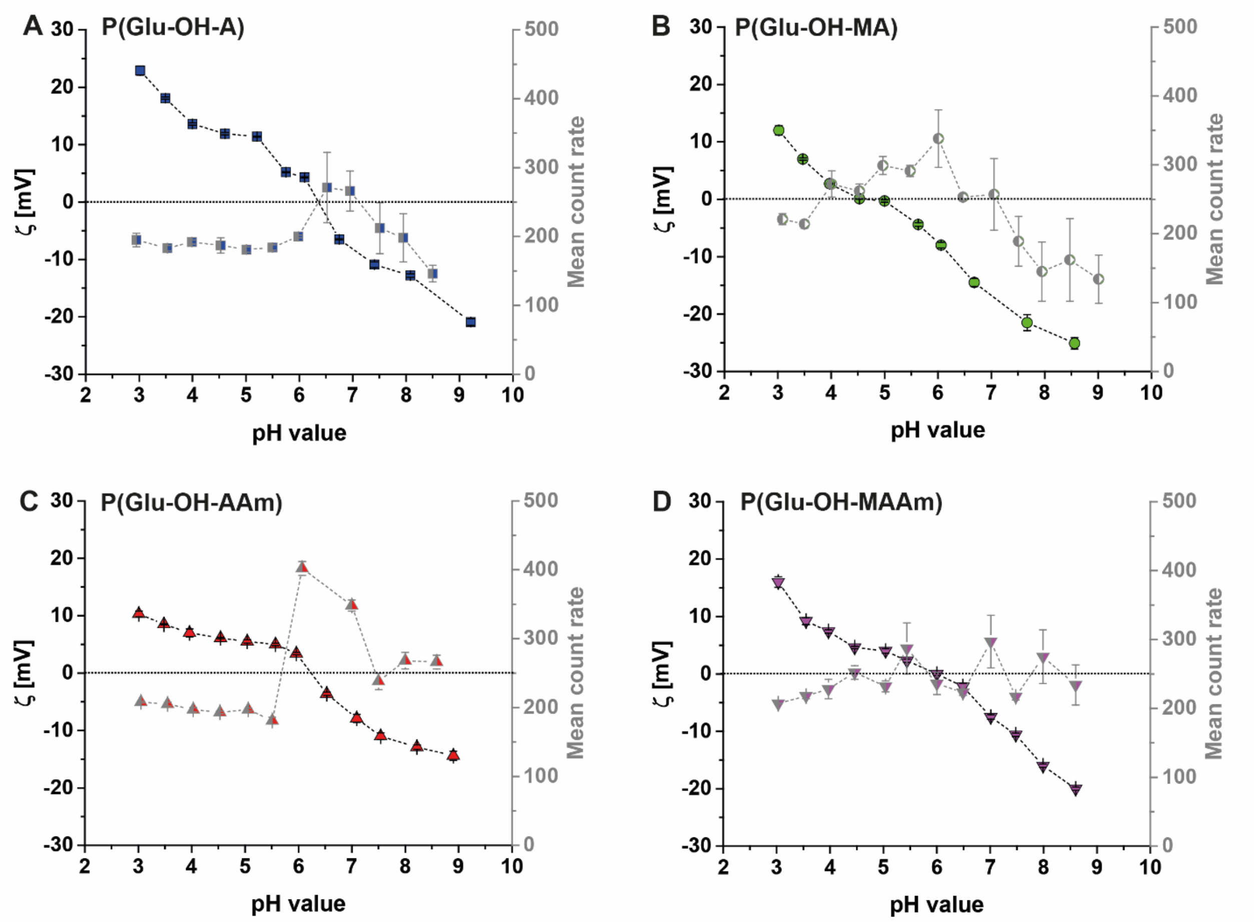

Figure S12. Zeta potential and mean count rate of different zwitterionic polymers determined by ELS and DLS measurements, respectively, in dependence of the pH value. Polymers were dissolved in diH_2O at a concentration of 10 mg mL^{-1} and the pH value was adjusted by the addition of 0.01 M NaOH solution. The dashed connecting line between the data points is added to guide the eye. Scatter represents the mean and SD of 3 measurements of the same solution. Dotted line indicates the isoelectric point (IEP).

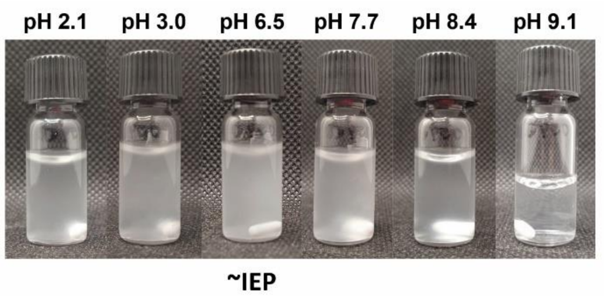

Figure S13. Turbidity of P(Glu-OH-$MAAm_{150}$) in diH_2O at indicated pH values. The polymer was dissolved in diH_2O at a concentration of 10 mg mL^{-1} and the pH value was adjusted by the addition of 0.01 M NaOH solution.

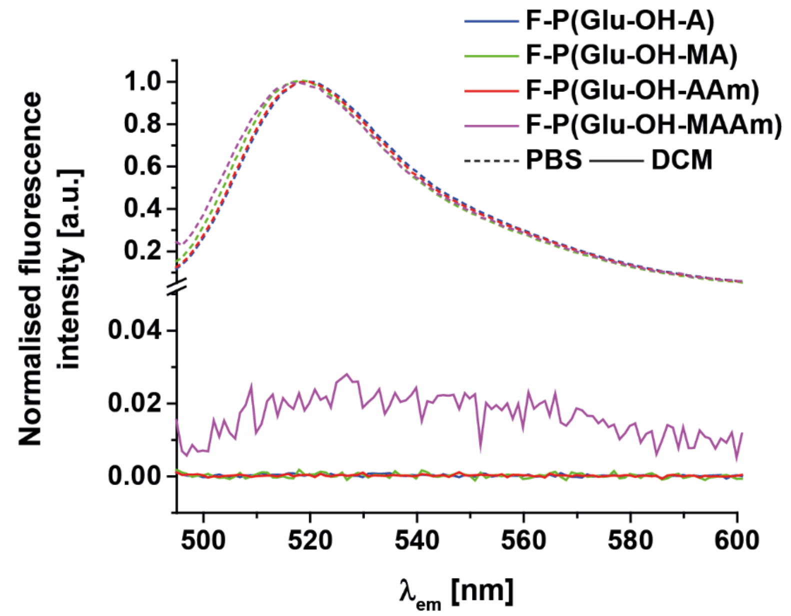

Figure S14. Hydrophilic-hydrophobic ratio of different Glu-derived polymers. Normalised fluorescence intensity was obtained by normalisation of the peak intensity by the peak maximum of the emission trace of the respective polymer observed in PBS (λ_{em} = 520 nm). λ_{ex} = 490 nm. Initial polymer concentration: 1 mg mL^{-1}.

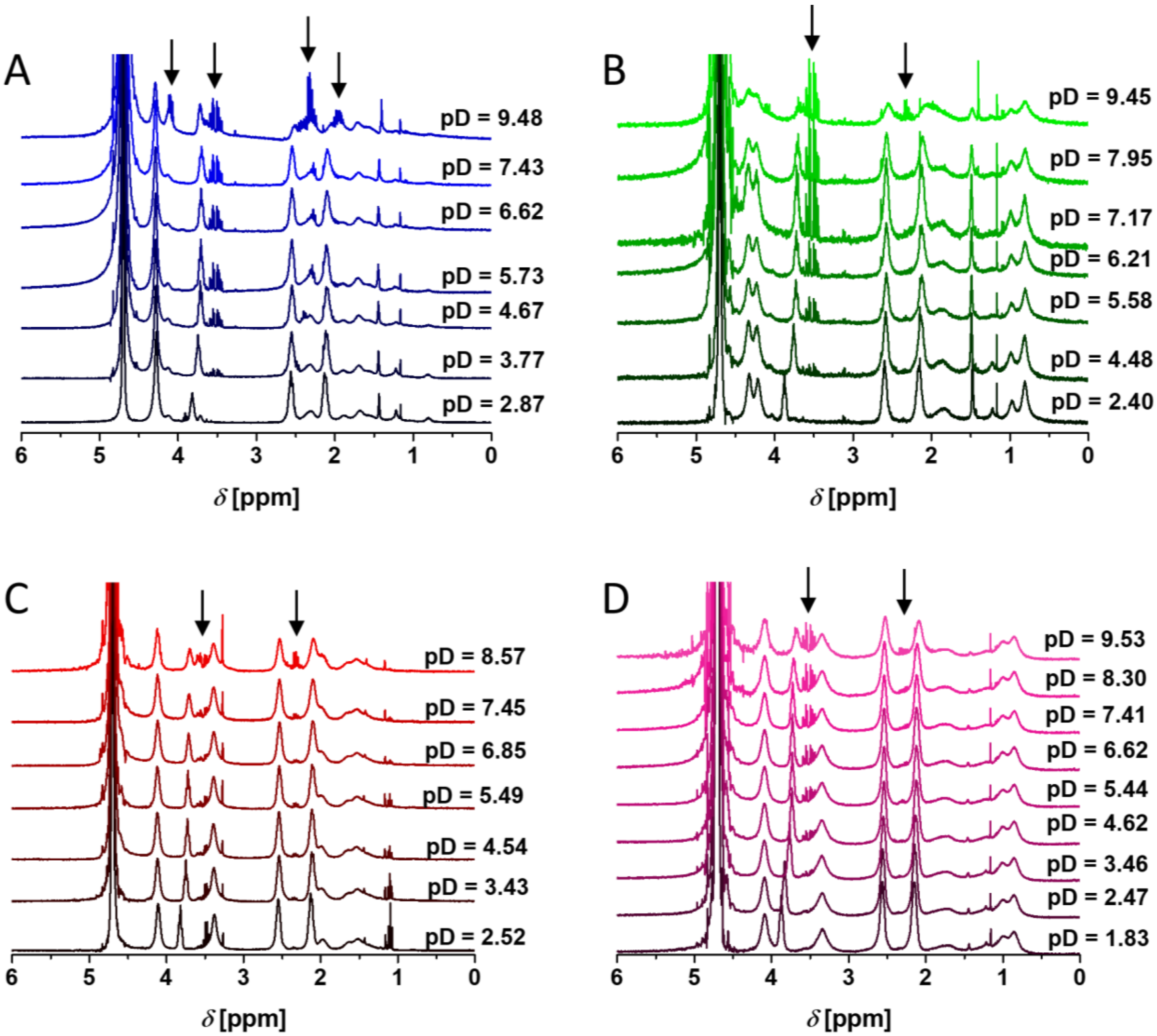

Figure S15. Characterisation of deprotected homopolymers at different pD values by ^{1}H NMR spectroscopy (300 MHz) in D_2O. Arrows indicate the appearance of degradation products, which were used to calculate the degree of degradation in Figure 2A. A: P(Glu-OH-A_{136}). B: P(Glu-OH-MA_{138}). C: P(Glu-OH-AAm_{140}). P(Glu-OH-$MAAm_{150}$). Polymers were dissolved in D_2O at a concentration of 10 mg mL^{-1} and the pH value was adjusted by the addition of 0.01 M NaOD solution. ^{1}H NMR measurements were performed immediately after pD adjustment.

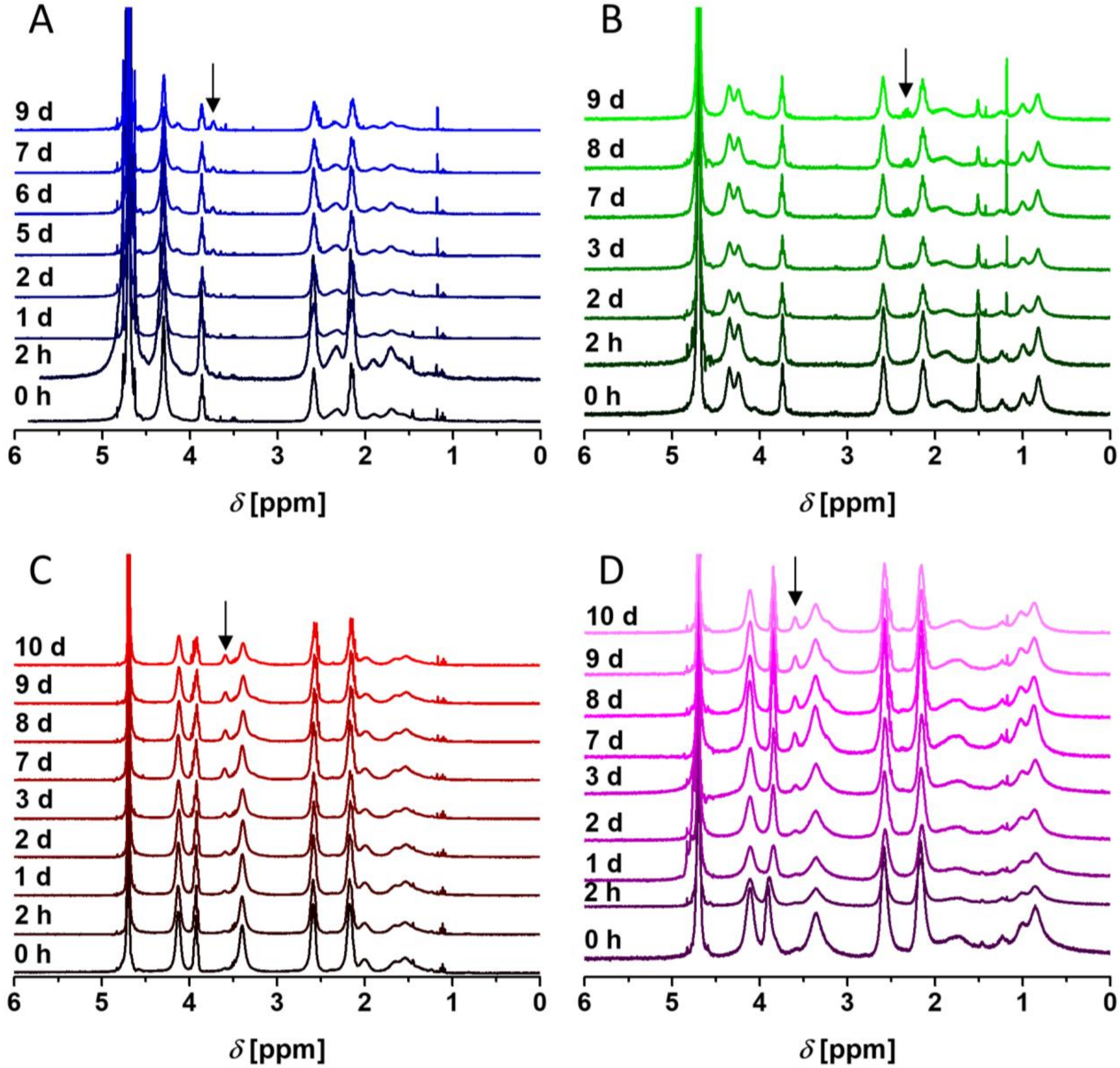

Figure S16. Characterisation of deprotected homopolymers after incubation in deuterated PBS at 37 °C for indicated times via ^{1}H NMR spectroscopy (300 MHz). Arrows indicate the appearance of degradation products, which were used to calculate the degree of degradation in Figure 2B. A: P(Glu-OH-A_{136}). B: P(Glu-OH-MA_{138}). C: P(Glu-OH-AAm_{140}). P(Glu-OH-$MAAm_{150}$). Polymers were dissolved in deuterated PBS at a concentration of 10 mg mL^{-1}. ^{1}H NMR measurements were performed at room temperature.

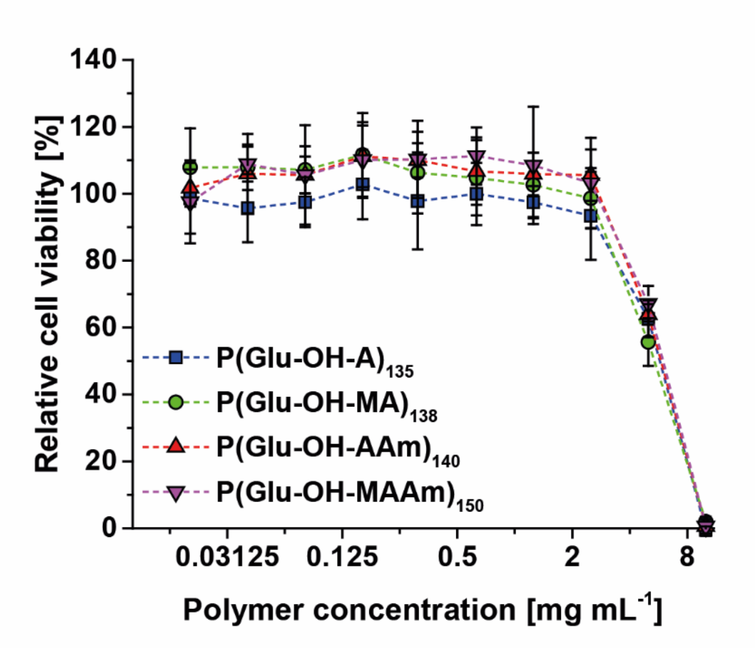

Figure S17. Cell viability of MDA-MB-231 breast cancer cells after incubation with various Glu-derived polymers at indicated concentrations for 24 h. Cell viability was determined by MTT assay. Cells without polymer treatment served as negative control (NC, 100% cell viability). Values shown are relative to the NC. Cells treated with 20% DMSO served as positive control (PC, 0% cell viability, data not shown).

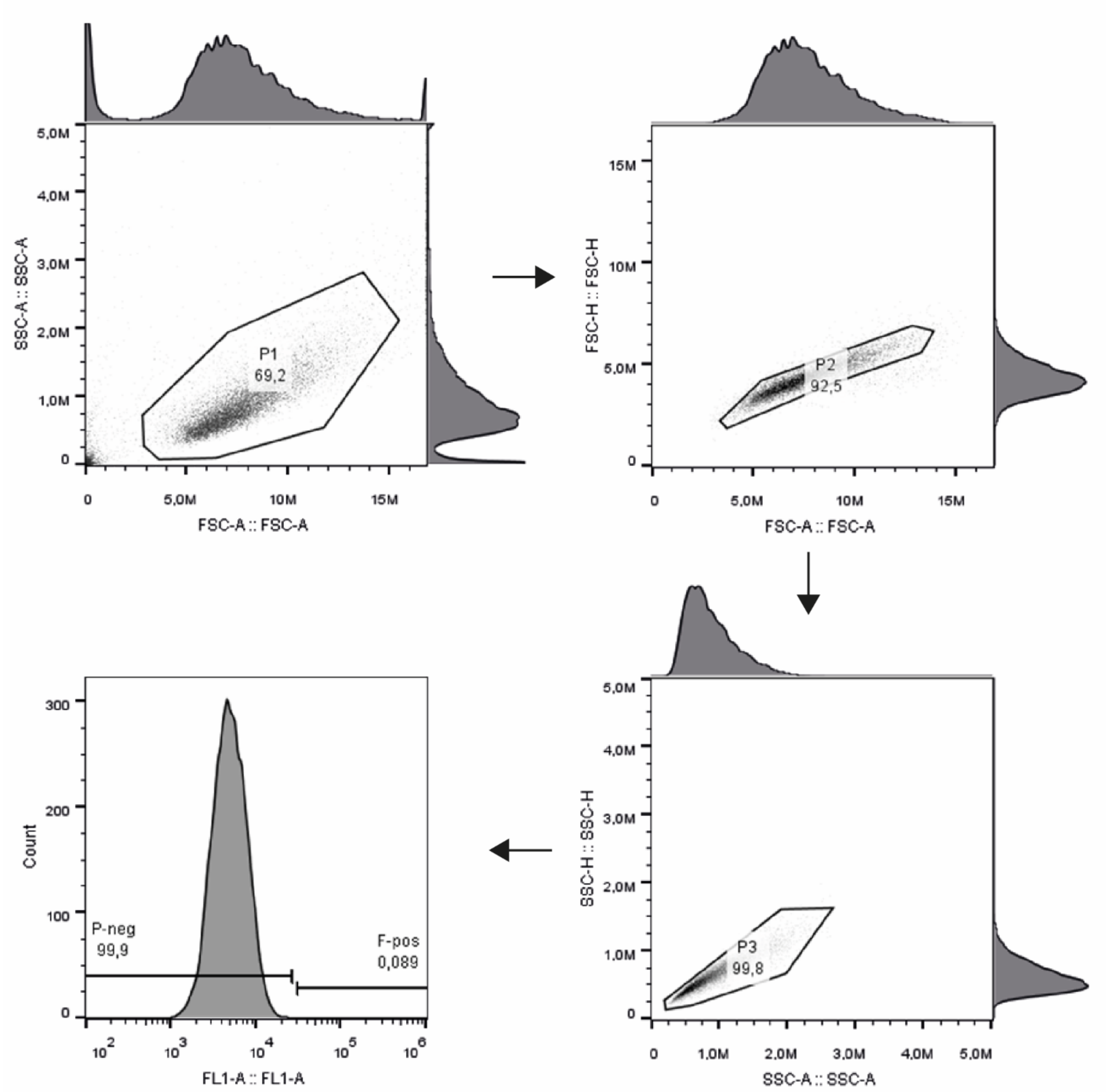

Figure S18. Representative gating strategy for MDA-MB-231. P1 corresponds to living cells, identified by the area of side scattering (SSC-A) and forward scattering (FSC-A). After wards, the height of forwards scattering (FSC-H) over FSC-A was applied on P1 to exclude cell doublets and identify P2 as population of single cells. Then, the height of side-scattering (SSC-H) was compared to SSC-A to identify P3 as the desired population of MDA-MB-231. Of this population, the mean fluorescence intensity (MFI) of the cells was determined at λ_{ex} = 488 nm (FL1-A). The MFI of control cells, which were incubated in the absence of polymer served as control to determine the autofluorescence of the cells.

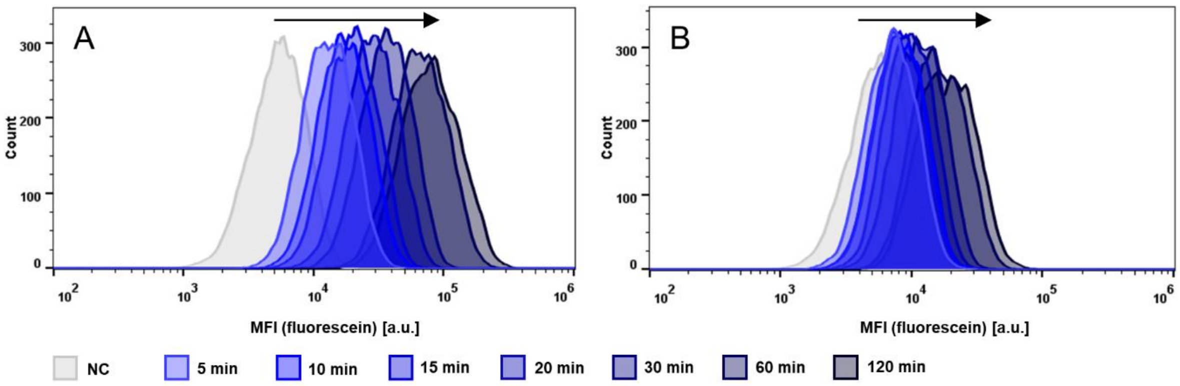

Figure S19. MFI histograms of time-dependent cell association of P(Glu-OH-A) with MDA-MB-231 at 37 °C for indicated time points. Polymer concentration: 0.1 mg mL^{-1}. 50,000 cells per well in 24-well plate. Arrow indicates increase in cell MFI. One representative sample of a triplicate is shown. Incubation in A: DMEM-F12 (no FBS) or B: DMEM-F12 supplemented with 10% FBS.

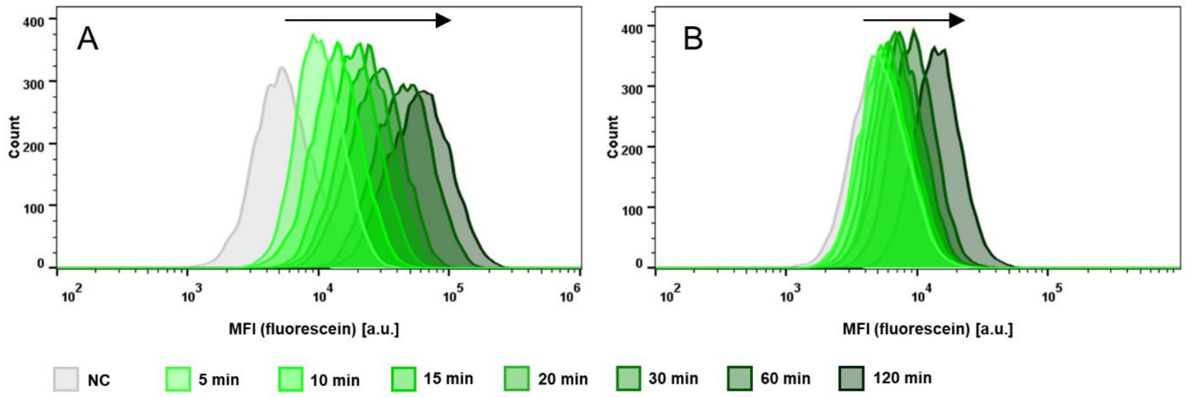

Figure S20. MFI histograms of time-dependent cell association of P(Glu-OH-MA) with MDA-MB-231 at 37 °C for indicated time points. Polymer concentration: 0.1 mg mL^{-1}. 50,000 cells per well in 24-well plate. Arrow indicates increase in cell MFI. One representative sample of a triplicate is shown. Incubation in A: DMEM-F12 (no FBS) or B: DMEM-F12 supplemented with 10% FBS.

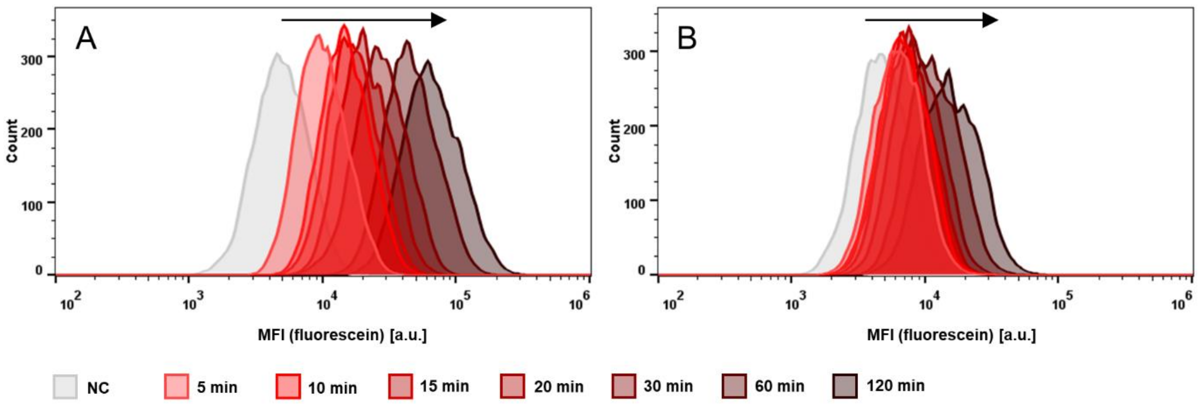

Figure S21. MFI histograms of time-dependent cell association of P(Glu-OH-AAm) with MDA-MB-231 at 37 °C for indicated time points. Polymer concentration: 0.1 mg mL^{-1}. 50,000 cells per well in 24-well plate. Arrow indicates increase in cell MFI. One representative sample of a triplicate is shown. Incubation in A: DMEM-F12 (no FBS) or B: DMEM-F12 supplemented with 10% FBS.

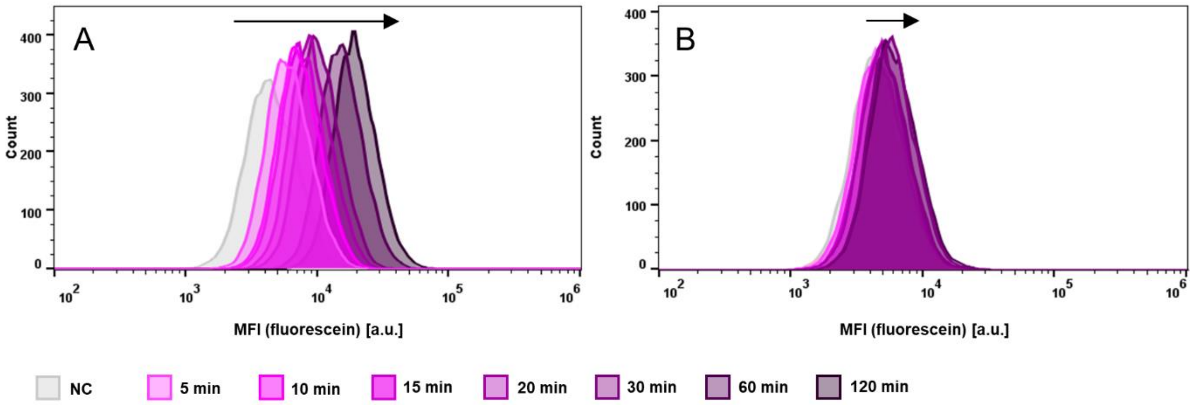

Figure S22. Raw MFI histograms of time-dependent cell association of P(Glu-OH-MAAm) with MDA-MB-231 at 37 °C for indicated time points. Polymer concentration: 0.1 mg mL^{-1}. 50,000 cells per well in 24-well plate. Arrow indicates increase in cell MFI. One representative sample of a triplicate is shown. Incubation in A: DMEM-F12 (no FBS) or B: DMEM-F12 supplemented with 10% FBS.

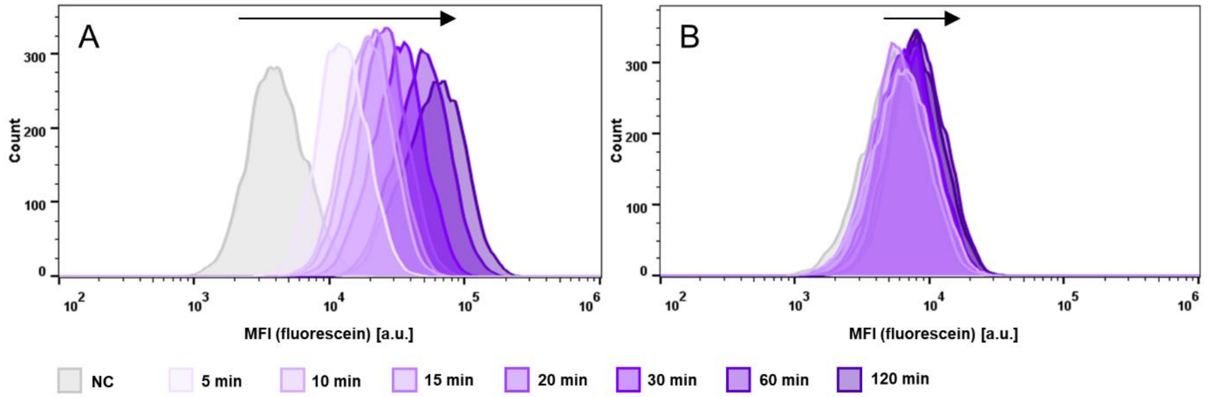

Figure S23. Raw MFI histograms of time-dependent cell association of POEGMA with MDA-MB-231 at 37 °C for indicated time points. Polymer concentration: 0.1 mg mL^{-1}. 50,000 cells per well in 24-well plate. Arrow indicates increase in cell MFI. One representative sample of a triplicate is shown. Incubation in A: DMEM-F12 (no FBS) or B: DMEM-F12 supplemented with 10% FBS.

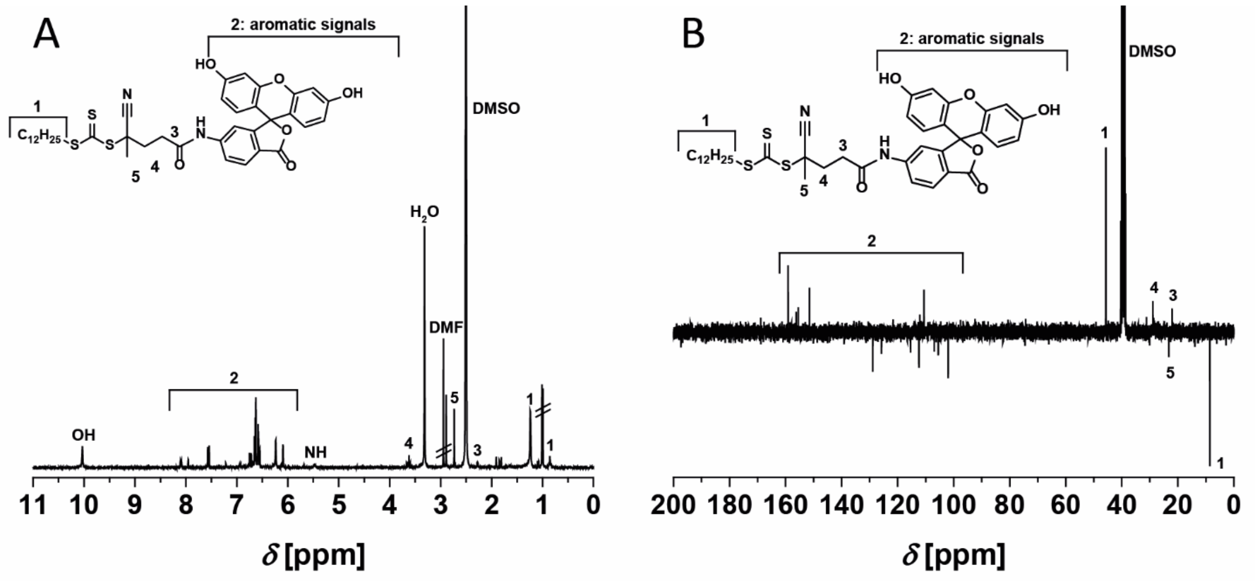

Figure S24. Characterisation of the 6AF-CTA. A: ^{1}H NMR spectrum (400 MHz, DMSO-d_6). B: ^{13}C (DEPT) NMR spectrum (100 MHz, DMSO-d_6).

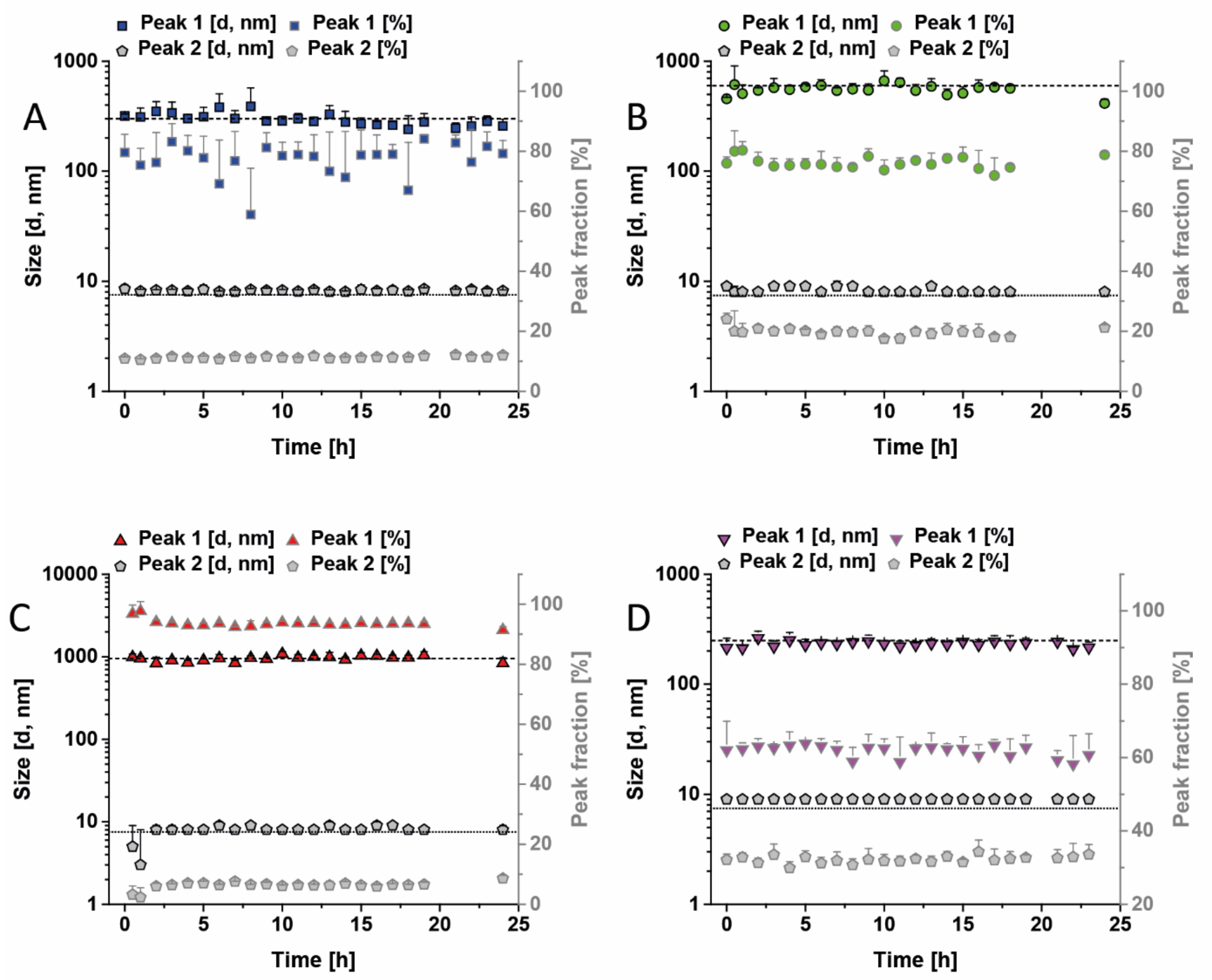

Figure S25. Interaction of zwitterionic polymers with BSA over time as determined by DLS measurements. Peak 1 corresponds to the polymer. Peak 2 corresponds to BSA. A: P(Glu-OH-A). B: P(Glu-OH-MA). C: P(Glu-OH-AAm). D: P(Glu-OH-MAAm).

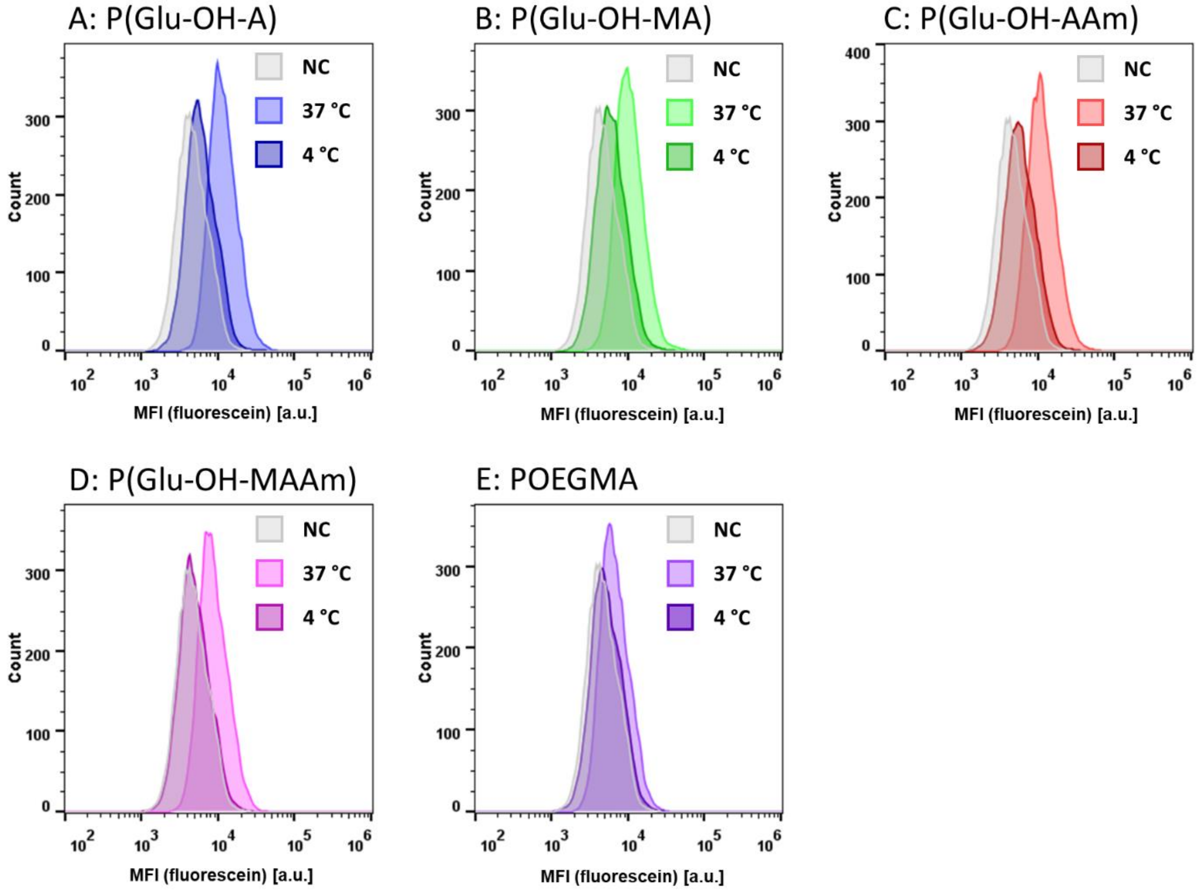

Figure S26. Raw MFI histograms of temperature-dependent cell association of different polymers with MDA-MB-231 breast cancer cells at 37 °C or 4 °C for 2 h. Polymer concentration: 0.1 mg mL^{-1}. 50,000 cells per well in 24-well plate. DMEM-F12 supplemented with 10% FBS. NC: Negative control.

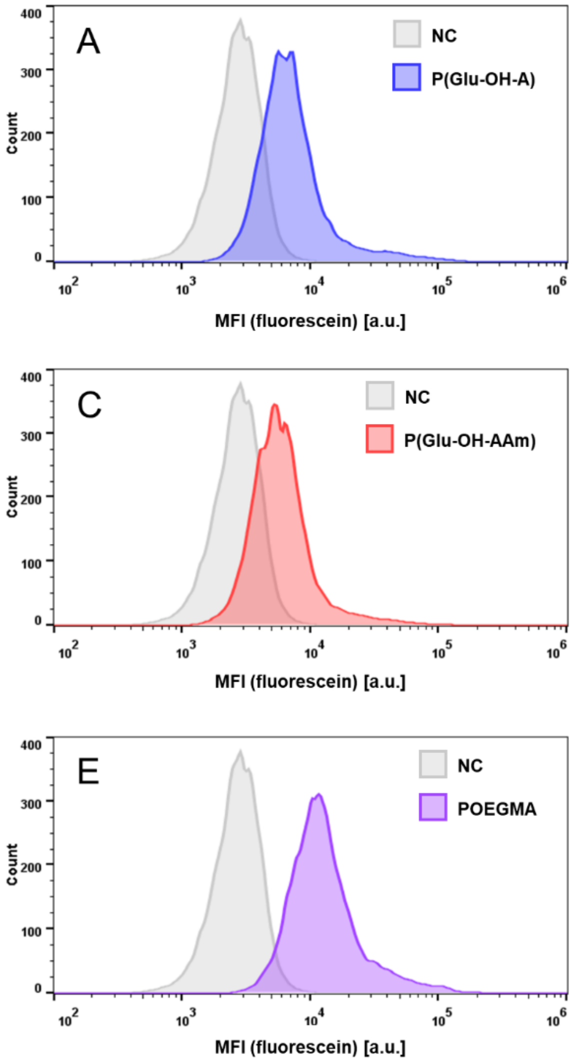

Figure S27. Raw MFI histograms of time-dependent cell association of different polymers with DC2.4 fibroblasts at 37 °C for 1 h. Polymer concentration: 0.1 mg mL^{-1}. 50,000 cells per well in 24-well plate. DMEM-F12 supplemented with 10% FBS. NC: Negative control.

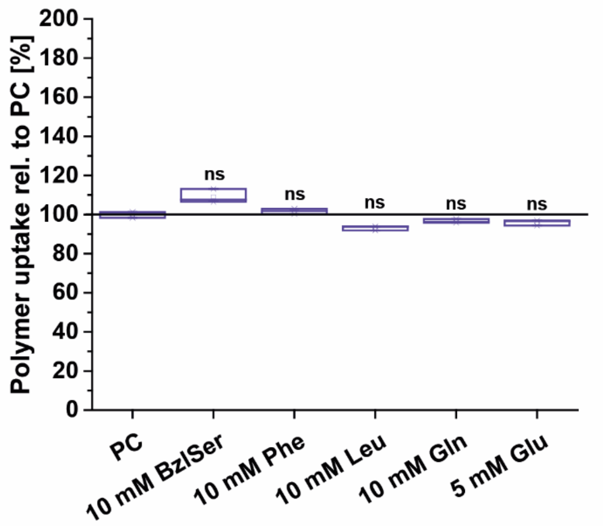

Figure S28. Relative cellular association of fluoresceine-labelled POEGMA with MDA-MB-231 breast cancer cells in the absence and presence of competitive amino acids determined via flow cytometry measurements. Polymer concentration: 0.1 mg mL^{-1}. Incubation for 1 h at 37 °C. 50,000 cells per well in 500 µL of DMEM-F12 + 10% FBS (24-well plate). Statistical significance was analysed by one-way ANOVA with Tukey's test. ns not significant at $p < 0.05$.

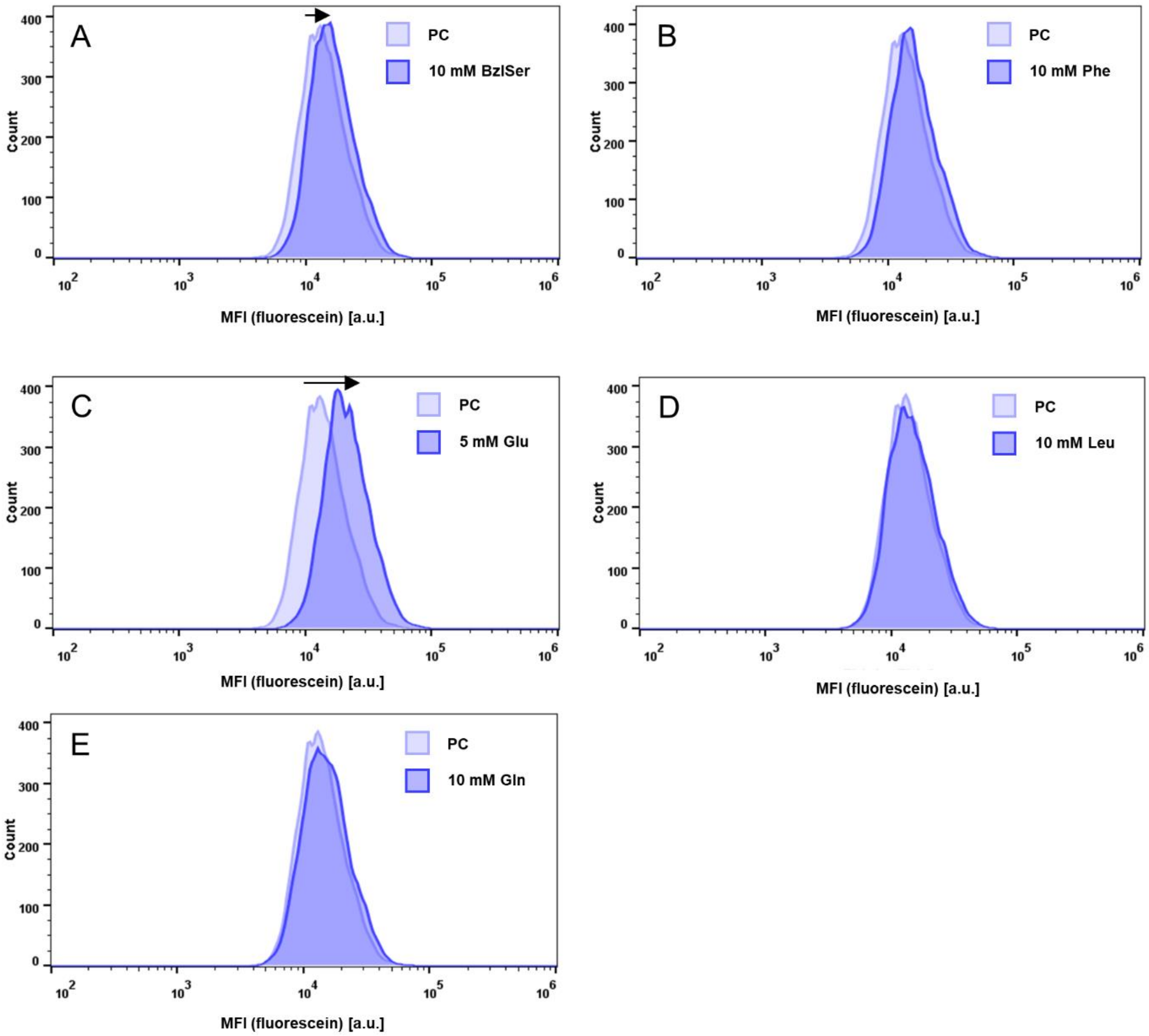

Figure S29. Raw MFI histograms of cell association of P(Glu-OH-A) with MDA-MB-231 in the absence (PC, positive control) or presence of competitive amino acids. Cells were incubated in DMEM-F12 supplemented with 10% FBS with or without indicated amino acids at 37 °C for 1 h. Polymer concentration: 0.1 mg mL^{-1}. 50,000 cells per well in 24-well plate. Arrow indicates increase or decrease in MFI. One representative sample of n = 9 is shown.

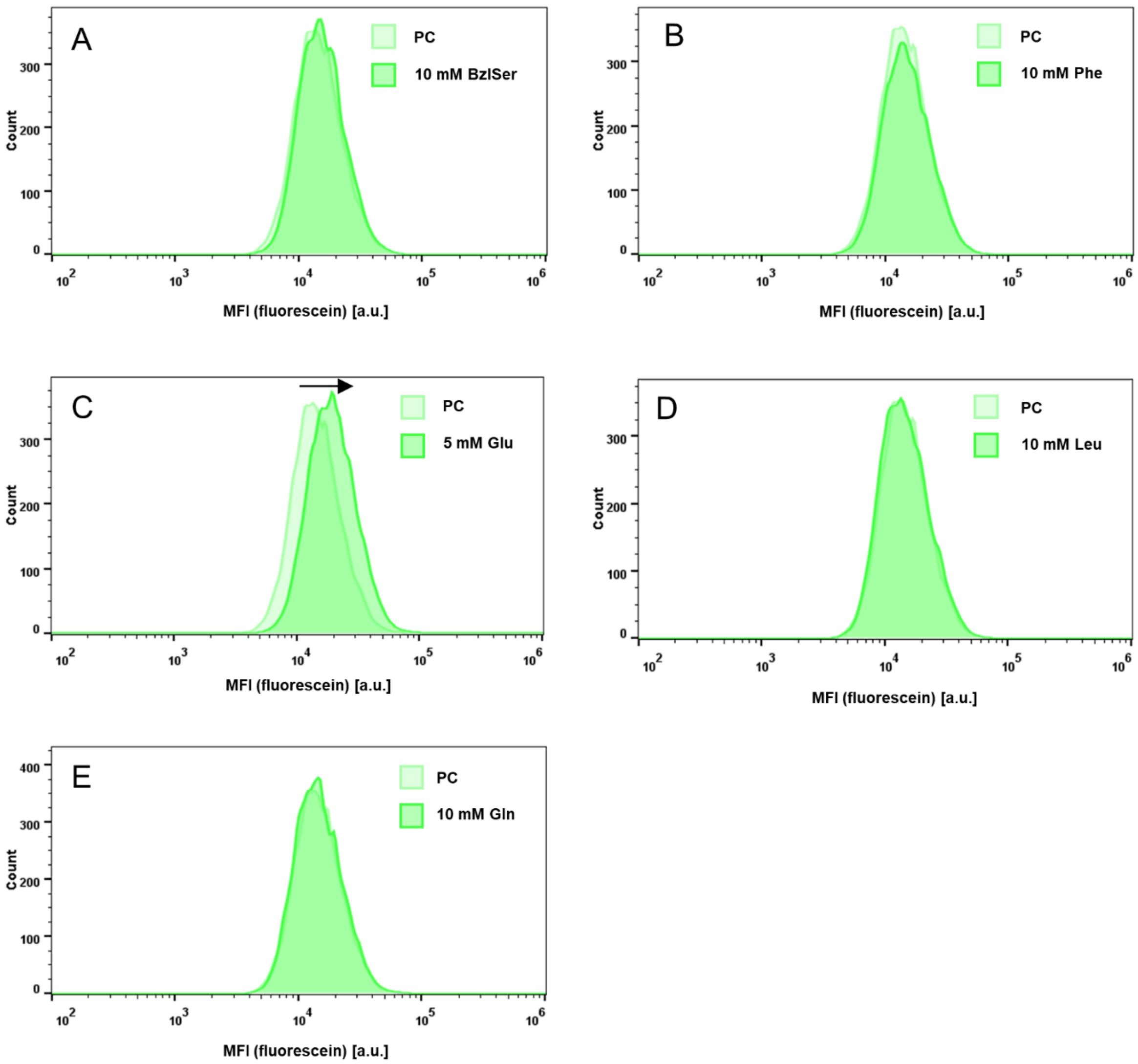

Figure S30. Raw MFI histograms of cell association of P(Glu-OH-MA) with MDA-MB-231 in the absence (PC, positive control) or presence of competitive amino acids. Cells were incubated in DMEM-F12 supplemented with 10% FBS with or without indicated amino acids at 37 °C for 1 h. Polymer concentration: 0.1 mg mL^{-1}. 50,000 cells per well in 24-well plate. Arrow indicates increase or decrease in MFI. One representative sample of n = 9 is shown.

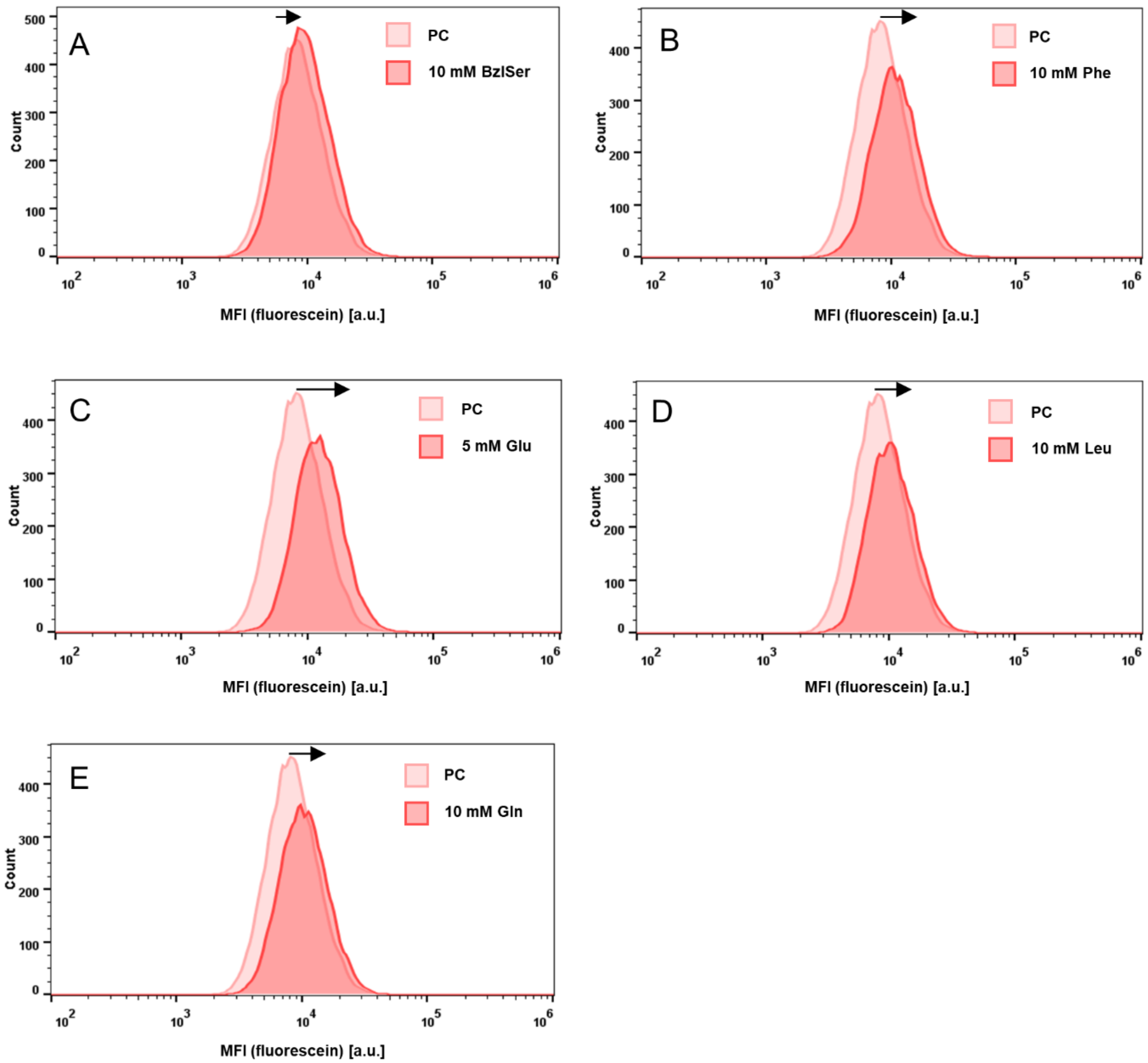

Figure S31. Raw MFI histograms of cell association of P(Glu-OH-AAm) with MDA-MB-231 in the absence (PC, positive control) or presence of competitive amino acids. Cells were incubated in DMEM-F12 supplemented with 10% FBS with or without indicated amino acids at 37 °C for 1 h. Polymer concentration: 0.1 mg mL^{-1}. 50,000 cells per well in 24-well plate. Arrow indicates increase or decrease in MFI. One representative sample of n = 9 is shown.

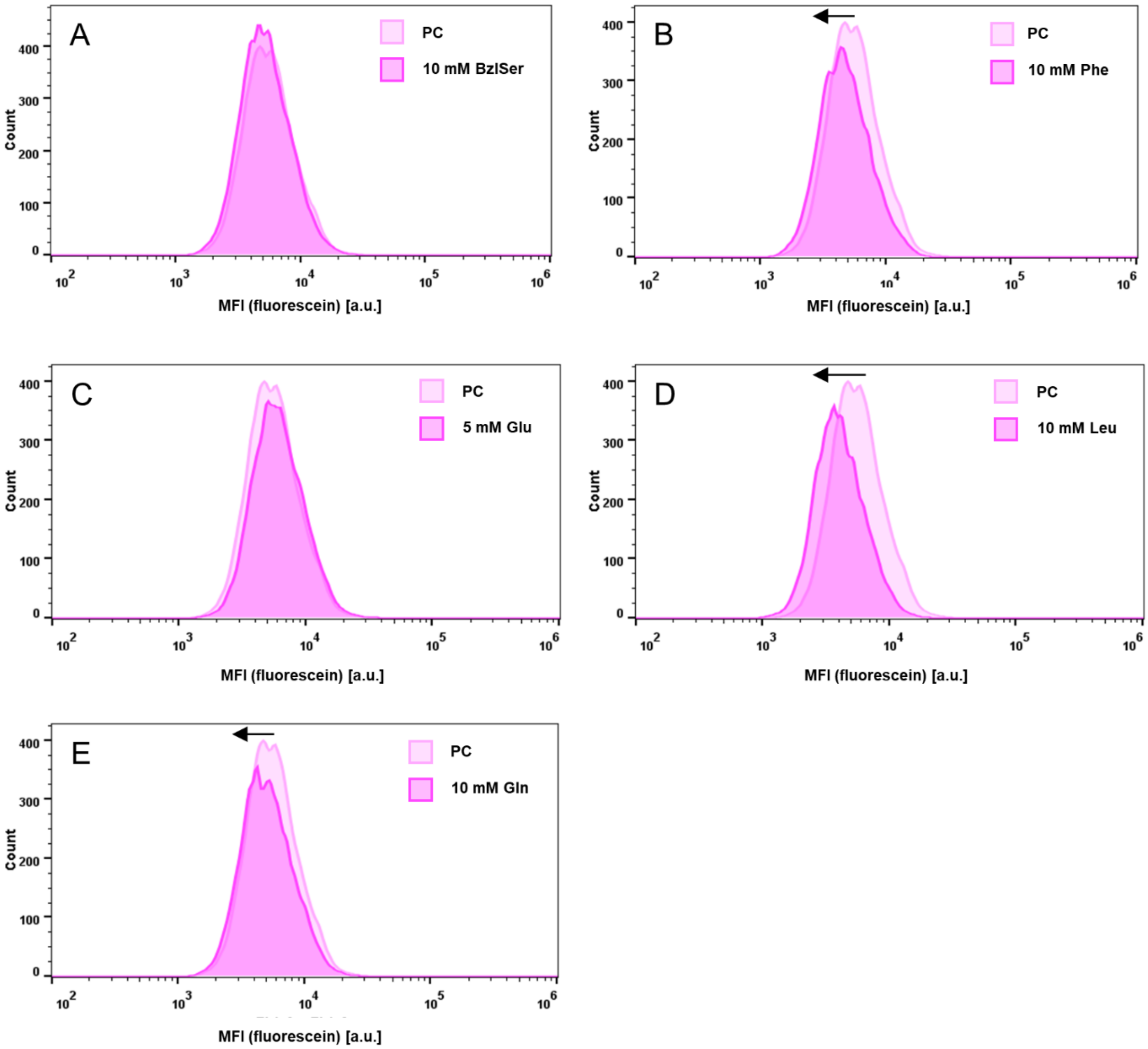

Figure S32. Raw MFI histograms of cell association of P(Glu-OH-MAAm) with MDA-MB-231 in the absence (PC, positive control) or presence of competitive amino acids. Cells were incubated in DMEM-F12 supplemented with 10% FBS with or without indicated amino acids at 37 °C for 1 h. Polymer concentration: 0.1 mg mL^{-1}. 50,000 cells per well in 24-well plate. Arrow indicates increase or decrease in MFI. One representative sample of n = 9 is shown.

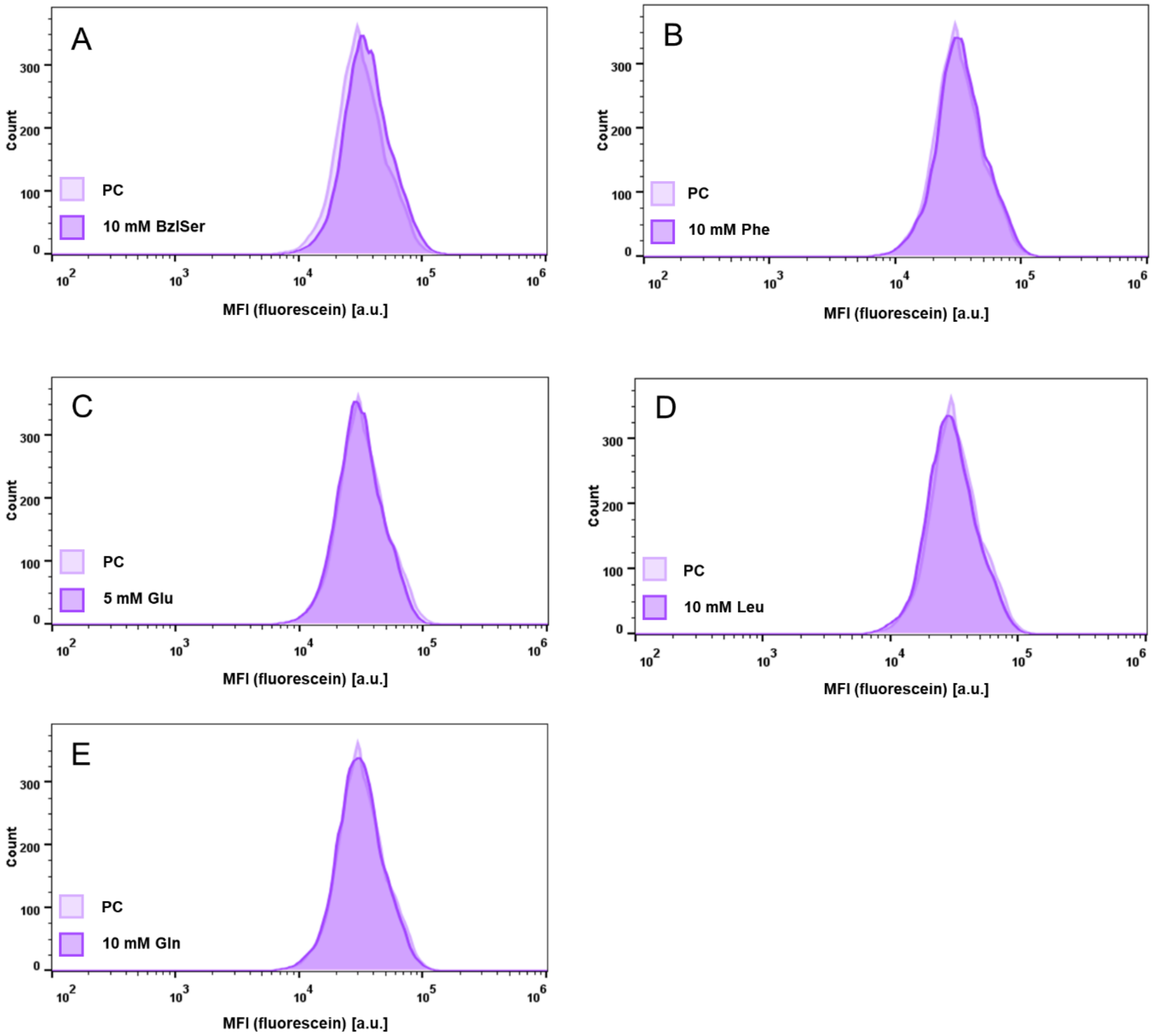

Figure S33. Raw MFI histograms of cell association of POEGMA with MDA-MB-231 in the absence (PC, positive control) or presence of competitive amino acids. Cells were incubated in DMEM-F12 supplemented with 10% FBS with or without indicated amino acids at 37 °C for 1 h. Polymer concentration: 0.1 mg mL^{-1}. 50,000 cells per well in 24-well plate. Arrow indicates increase or decrease in MFI. One representative sample of n = 3 is shown.

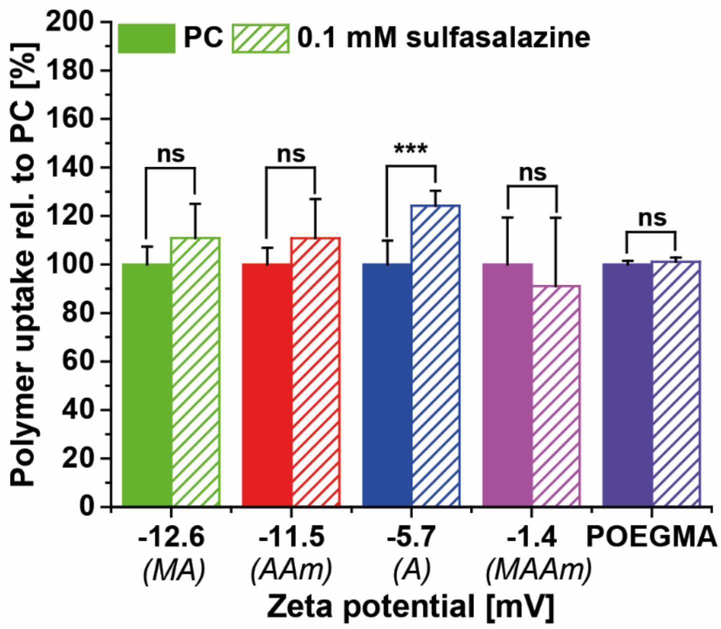

Figure S34. Relative cellular association of different fluoresceine-labelled zwitterionic polymers with MDA-MB-231 breast cancer cells in the absence (positive control, PC) and presence of sulfasalazine (c = 0.1 mM) determined *via* flow cytometry measurements. Polymer concentration: 0.1 mg mL^{-1}. Incubation for 1 h at 37 °C. 50,000 cells per well in 500 µL of DMEM/F12 + 10% FBS (24-well plate). Statistical significance was analysed by one-way ANOVA with Tukey's test. ***$p < 0.0005$; ns not significant at $p < 0.05$.

Table S1. Characterisation of polymers.

	^1H NMR[a]			SEC			
Polymer	Conv. [%]	DP_{theo}[b] (conv.)	$M_{n, theo}$[b] [kg mol^{-1}]	M_n (RI)[c] [kg mol^{-1}]	Đ (RI)[c]	M_n (LS)[d] [kg mol^{-1}]	Đ[d] (LS)
P(*N*Boc-Glu-O*t*Bu-A)-CTA	90	136	54.6	27.8	1.20	n.d.	n.d.
P(*N*Boc-Glu-O*t*Bu-MA)-CTA	92	138	57.3	36.1	1.26	n.d.	n.d.
P(*N*Boc-Glu-O*t*Bu-AAm)-CTA	93	140	56.1	46.0	1.34	n.d.	n.d.
P(*N*Boc-Glu-O*t*Bu-MAAm)-CTA	100	150	62.3	59.9	1.37	n.d.	n.d.
P(*N*Boc-Glu-O*t*Bu-A)	n.a.	136	54.4	23.2	1.44	39.8	1.23
P(*N*Boc-Glu-O*t*Bu-MA)	n.a.	138	57.1	39.4	1.23	55.0	1.19
P(*N*Boc-Glu-O*t*Bu-AAm)	n.a.	140	55.9	46.4	1.30	55.8	1.23
P(*N*Boc-Glu-O*t*Bu-MAAm)	n.a.	150	62.1	63.1	1.32	69.7	1.26
P(Glu-OH-A)	n.a.	136	33.4	6.5[e]	1.65[e]	n.d.	n.d.
P(Glu-OH-MA)	n.a.	138	35.8	13.1[e]	1.36[e]	n.d.	n.d.
P(Glu-OH-AAm)	n.a.	140	34.2	10.4[e]	1.34[e]	n.d.	n.d.
P(Glu-OH-MAAm)	n.a.	150	38.7	6.7[e]	1.53[e]	n.d.	n.d.

[a] ^1H NMR (300 MHz) in $CDCl_3$. [b]calculated from monomer conversion. [c]SEC in DMAc (PMMA calibration). [d]SEC in DMAc (MALS). [e]SEC in acetate buffer at pH 3.6 containing 30% (v/v) MeCN and 0.1 M $NaNO_3$ (PEG calibration).

Table S2. Properties of zwitterionic polymers in aqueous environments.

Polymer	IEP[a] (diH_2O)	ZP[a] [mV] (DMEM/F12)	CC_{50}[b] [mg mL^{-1}]
P(Glu-OH-A)$_{135}$	6.4	-5.7 ± 0.3	5.7
P(Glu-OH-MA)$_{138}$	4.8	-12.6 ± 0.7	5.4
P(Glu-OH-AAm)$_{140}$	6.2	-11.5 ± 0.5	5.8
P(Glu-OH-MAAm)$_{150}$	6.0	-1.4 ± 0.3	6.0

[a]Determined by ELS measurements (see Figure S10).
[b]Determined by MTT assay (see Figure S16).

References

[1] H. Ohshima, *J. Colloid Interface Sci.*, **1994**, *168*, 269-271. doi: 10.1006/jcis.1994.1419

[2] M. N. Leiske, Z. A. I. Mazrad, A. Zelcak, K. Wahi, T. P. Davis, J. A. McCarroll, J. Holst and K. Kempe, *Biomacromolecules*, **2022**, *23*, 2374-2387. doi: 10.1021/acs.biomac.2c00143

[3] M. C. Spiridon, F. A. Jerca, V. V. Jerca, D. M. Vuluga and D. S. Vasilescu, *Sci Bull Politeh Univ Buchar Ser B*, **2014**, *76*, 59-70.

[4] M. Chen, G. Moad and E. Rizzardo, *J. Polym. Sci., Part A: Polym. Chem.*, **2009**, *47*, 6704-6714. doi: 10.1002/pola.23711
